# Sprouts and Microgreens—Novel Food Sources for Healthy Diets

**DOI:** 10.3390/plants11040571

**Published:** 2022-02-21

**Authors:** Andreas W. Ebert

**Affiliations:** World Vegetable Center, 60 Yi-Min Liao, Shanhua, Tainan 74151, Taiwan; ebert.andreas6@gmail.com

**Keywords:** microscale vegetables, sprouts, microgreens, phytonutrients, functional foods, malnutrition, seed priming, biofortification, illumination, health-promoting compounds

## Abstract

With the growing interest of society in healthy eating, the interest in fresh, ready-to-eat, functional food, such as microscale vegetables (sprouted seeds and microgreens), has been on the rise in recent years globally. This review briefly describes the crops commonly used for microscale vegetable production, highlights *Brassica* vegetables because of their health-promoting secondary metabolites (polyphenols, glucosinolates), and looks at consumer acceptance of sprouts and microgreens. Apart from the main crops used for microscale vegetable production, landraces, wild food plants, and crops’ wild relatives often have high phytonutrient density and exciting flavors and tastes, thus providing the scope to widen the range of crops and species used for this purpose. Moreover, the nutritional value and content of phytochemicals often vary with plant growth and development within the same crop. Sprouted seeds and microgreens are often more nutrient-dense than ungerminated seeds or mature vegetables. This review also describes the environmental and priming factors that may impact the nutritional value and content of phytochemicals of microscale vegetables. These factors include the growth environment, growing substrates, imposed environmental stresses, seed priming and biostimulants, biofortification, and the effect of light in controlled environments. This review also touches on microgreen market trends. Due to their short growth cycle, nutrient-dense sprouts and microgreens can be produced with minimal input; without pesticides, they can even be home-grown and harvested as needed, hence having low environmental impacts and a broad acceptance among health-conscious consumers.

## 1. Introduction

Healthy diets are essential for nutrition and health [1]. As defined by Neufeld et al. [2], a healthy diet is “health-promoting and disease-preventing. It provides adequacy without excess, of nutrients and health-promoting substances from nutritious foods and avoids the consumption of health-harming substances.” About three billion people cannot afford healthy diets around the globe. This figure includes most people living in sub-Saharan Africa and South Asia [3,4]. The Sustainable Development Goal 2 (SDG 2) ‘Zero Hunger’ of the United Nations calls for the eradication of hunger and all forms of malnutrition. All people ought to have access to safe, nutritious, and sufficient food all year round by 2030 [5]. The triple burden of malnutrition, i.e., undernutrition, micronutrient deficiency, and overnutrition, affects most nations around the globe. As incomes rise and food consumption patterns change, overnutrition from imbalanced diets increasingly becomes a concern in developed and developing countries.

Malnutrition is a high-risk factor for non-communicable diseases (NCDs), also known as chronic diseases. Diet-related NCDs, such as diabetes, cardiovascular disease, hypertension, stroke, cancer, and obesity, are escalating globally. Out of the estimated 40.5 million people killed by NCDs each year (71% of the annual deaths worldwide), approximately 32.2 million NCD deaths (80%) were attributable to cancers, cardiovascular diseases, chronic respiratory diseases, and diabetes [6,7]. The remaining 8.3 million NCD deaths (20%) have other root causes. These figures illustrate the seriousness of diet-related diseases for the healthcare sector. Under SDG 3—‘Good Health and Well-Being’—SDG target 3.4 aims at reducing premature mortality from NCDs by one-third by 2030 [5]. The diversity and quality of food produced sustainably and made accessible to a wide range of consumers are decisive factors that enable substantial dietary shifts [8,9] and, in turn, help to address SDG targets 2.1 and 3.4. A truly transformed global food system may not only provide universal access to healthy diets but may also co-deliver on climate and environmental SDGs [10].

The nutrition community frequently highlights the importance of fruits, vegetables, and nuts in combating the triple burden of malnutrition [11]. The British Royal Society’s strategy to eliminate hidden hunger involves measures that promote increased access to fruits and vegetables to enhance dietary diversity [12]. The World Health Organization (WHO) recommends a population-wide daily intake of 400 g of edible fruits and vegetables to prevent NCDs and alleviate several micronutrient deficiencies [13]. This WHO recommendation translates to roughly five portions of fruits and vegetables per day. People able to enjoy more diverse diets, in general, also have better nutrition and health. A recent study analyzing data of a health survey in Great Britain revealed a robust inverse association between fruit and vegetable consumption and mortality [14].

Despite this general acceptance that fruits and vegetables are essential for a healthy diet, the authors of several studies concluded that current and projected fruit and vegetable production levels would fail to meet healthy consumption levels [15,16]. Based on age-specific recommendations, only 40 countries representing 36% of the global population had adequate availability of fruits and vegetables in 2015 [17]. Although there was a sharp increase in vegetable consumption in sub-Saharan Africa over the last three decades, the combined fruit and vegetable intake (268 g) remains well below the WHO recommendation of 400 g [18].

With society’s growing interest in healthy eating and lifestyles, e.g., the Slow Food movement and the promotion of novel and superfoods, the interest in fresh, ready-to-eat functional and nutraceutical food has been on the rise in recent decades [19,20]. In this context, microscale vegetables, i.e., sprouted seeds and microgreens, are becoming increasingly popular worldwide as fresh, ready-to-eat functional and nutraceutical food. They have great potential to diversify and enhance the human diet and address nutrient deficiencies due to their high content of phytochemicals [21,22,23,24,25,26].

Sprouts are commonly grown in the dark under high relative humidity. They are harvested when the cotyledons are still under-developed and true leaves have not begun to emerge, usually after 3–5 days from seed hydration. The entire plant (root, seed, and shoot) is consumed. Ancient Egyptians have already practiced sprouting seeds around 3000 B.C. [27]. During the germination process, the amount of antinutritive compounds (trypsin inhibitor, phytic acid, pentosan, tannin, and cyanides) decreases, while palatability and nutrient bioavailability, as well as the content of health-related phytochemicals (glucosinolates and natural antioxidants), are enhanced [28,29,30]. While sprouts usually take less than a week to mature, microgreens are harvested for consumption within 10–20 days of seedling emergence [31]. Microgreens, defined as tender, immature greens, are larger than sprouts, but smaller than baby vegetables or greens. They have a central stem with two fully developed, non-senescent cotyledon leaves and mostly one pair of small true leaves [21,32,33,34,35]. The stem, cotyledons, and first true leaves are consumed. Microgreens have been produced in Southern California since the 1980s [36,37] and have since gained popularity due to their vivid colors (like red and purple), delicate textures, and flavor-enhancing properties. They are used as garnishes in salads, sandwiches, soups, appetizers, desserts, and drinks [19] and are highly appreciated because of their nutritional benefits [21,22,23,24,25,26]. In vitro and in vivo research studies have demonstrated microgreens’ anti-inflammatory, anti-cancer, anti-bacterial, and anti-hyperglycemic properties, further strengthening their attractiveness as a new functional food that is beneficial to human health (see, e.g., review by Zhang et al. [37]).

Commercial and home-grown microgreen production comprises several aspects: selecting appropriate species, growing systems, substrates, quality of seeds, seeding and germination, irrigation and fertilization, harvesting, phytosanitary quality, and post-harvest storage practices. Di Gioia et al. [32] provided a detailed insight into these aspects. For a recent review of microgreen product types and production practices, readers may also consult Verlinden [38].

This comprehensive review describes the main crops used in microscale vegetable production and the factors that impact sprouts’ and microgreens’ nutritional and bioactive profile and their consumer acceptance. It also reflects on underutilized species (landraces, wild food plants, and crops’ wild relatives) that offer the scope to widen the range of crops used for this purpose. In addition, this paper reviews the effects of plant growth stages on the nutritional and bioactive composition of edible plant parts.

## 2. Crops Commonly Used for Microscale Vegetable Production

**Figure 1:**
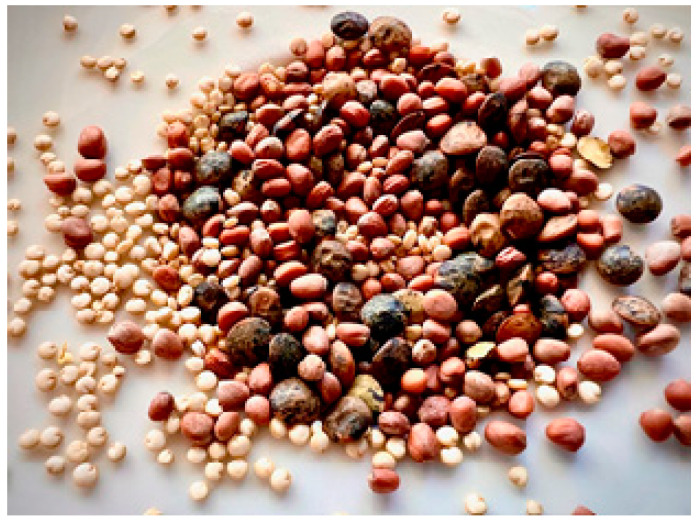
Dormant seed with stored reserves. A display of a seed mix for sprouting, consisting of quinoa (*Chenopodium quinoa*), lentil (*Lens culinaris*), and radish (*Raphanus sativus*) seed.

**Figure 2:**
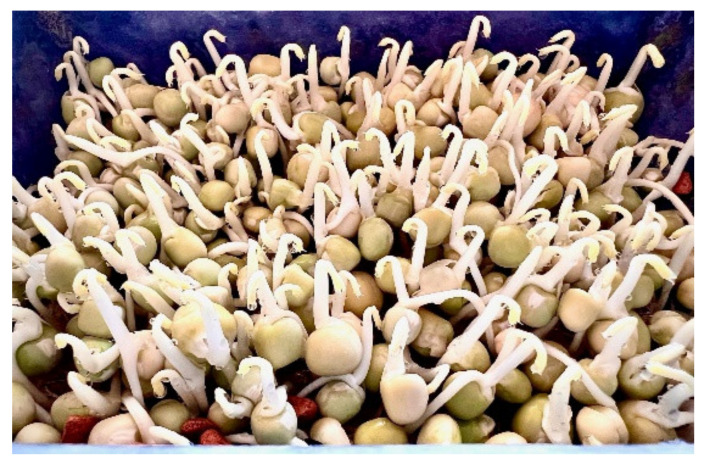
Home-grown 3-day old pea (*Pisum sativum*) sprouts. Seed germination process and nutritional benefits of sprouted seeds:

**Figure 3:**
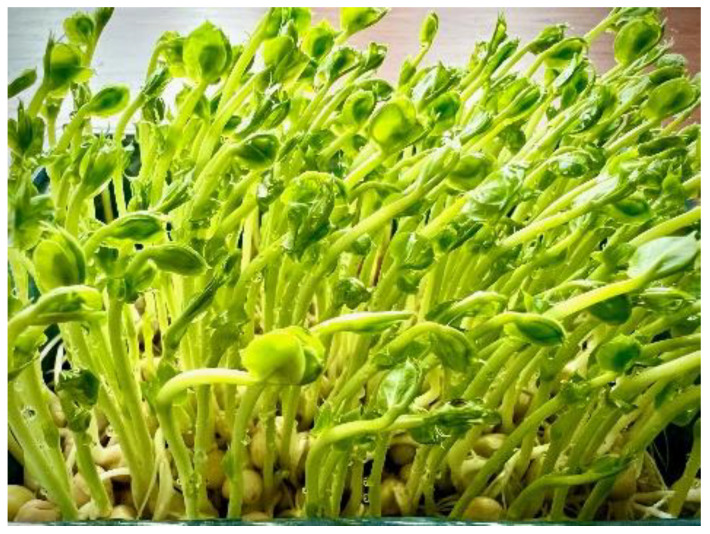
Home-grown 9-day old pea (*Pisum sativum*) microgreens. Commonly recognized nutritional benefits of microgreens:

Sprouts and microgreens are grown from the seeds of many crops, such as legumes, cereals, pseudo-cereals, oilseeds, vegetables, and herbs [21,38]; Table 1. The significant traits of interest for consumers are the appearance, texture, flavor, phytochemical composition, and nutritional value of sprouts and microgreens [39]. Most crops are grown for sprouts and microgreens, except for beans and some oilseed tree species that are commonly grown as sprouts only (Table 1). Mungbean and soybean sprouts have long been an essential, year-round component of Asian and vegetarian dishes [21,40]. In recent decades, mungbean sprouts have become increasingly popular in the Americas, Europe, and Africa. They are commonly recognized as “bean sprouts,” although this group comprises several different crops (see Table 1). Most food and forage legumes are known for their high nutritional value and an abundance of minerals and secondary metabolites. Sprouted seeds and microgreens often contain higher concentrations of bioactive compounds than raw seeds (Figure 1, Figure 2 and Figure 3) [41,42]. Sprouting cereal grains enhances their nutritional value, especially when applying a sprouting duration of at least 3 to 5 days [43]. The sprouting process activates hydrolytic enzymes and releases nutrients from their phytate chelates, making them bioavailable; in addition, vitamins are synthesized and accumulate [43]. Sprouted grains are also used in many staple foods such as bread, pasta, noodles, and breakfast flakes, but food processing often compromises their nutritional value.

Pseudocereals are underutilized food crops that are receiving increasing attention as highly nutritious and functional foods [44]. Among those, amaranth, quinoa, and buckwheat are increasingly becoming popular for sprout and microgreen production [45,46,47,48]. Apart from soybean, peanut (listed under legumes), and mustard (classified here as a vegetable), almond, hazelnut, linseed, sesame, and sunflower are other oilseed crops that one can use for sprouting or microgreen production (Table 1). Among the group of vegetables and herbs, members of the Brassicaceae family are widely used for sprouting and microgreen production, followed by crops of the Apiaceae, Fabaceae, and Amaranthaceae families (Table 1).

- Seed activation through imbibition, favourable temperature, oxygen, light, or darkness
- Enhanced respiration and metabolic activities
- Enzymes mobilize stored seed reserves and convert starch to sugar
- Hydrolysis of storage proteins, release of essential amino acids
- Accumulation of phenolic compounds with antioxidant ability
- Accumulation of vitamins (C, folate, thiamin, pyridoxin, tocopherols, niacin, etc.).

Reduction of antinutritional factors:

- Phytate, oxalate, and tannin degradation, leading to enhanced palatability, improved bioaccessibility of iron and calcium, and enhanced digestibility of proteins.

- Photosynthetic activity in microgreens further enhances vitamin C, phylloquinone, and tocopherol accumulation compared to sprouts
- Accumulation of carotenoids is often higher than in mature vegetables
- Increased accumulation of chlorophyll and phenolic compounds with antioxidant ability, compared to sprouts
- Often higher content of macro- and micronutrients and lower content of nitrate in microgreens compared to the adult growth stage
- Biofortification with specific elements (iodine, iron, zinc, selenium) made easy in hydroponic systems
- Microgreens are consumed raw, hence thermolabile ascorbic acid content can be fully utilized, unlike in cooked mature vegetables.

### 2.1. Bioactive Composition and Potential Health Effects of Brassica Microscale Vegetables

As evident from Table 1, the Brassicaceae family comprises a wide range of crops commonly used for microscale vegetable production. The intrinsic qualities of *Brassica* vegetables, including their color, aroma, taste, and health properties, are profoundly determined by secondary plant metabolite profiles and their concentrations in plant tissues [49]. *Brassica* vegetables are rich sources of bioactive compounds, such as glucosinolates (GSLs), polyphenols, anthocyanins, ascorbic acid, carotenoids, and tocopherols [50,51,52,53]. The biosynthesis of secondary plant metabolites is closely linked to plant protection and defense mechanisms and can be modulated by environmental and agronomical factors. Those factors may significantly change the concentration of secondary plant metabolites with up to 570-fold increases for specific compounds, such as isothiocyanates [49].

Among the bioactive compounds of *Brassica* vegetables, polyphenols and GSLs have been widely studied due to their known health-promoting effects [54,55], including the impact of cooking methods on the retention of these essential compounds [56]. In addition, polyphenols are good sources of natural antioxidants, which help decrease the risk of diseases associated with oxidative stress [57]. GSLs, defined as aliphatic, aromatic, or indolic based on their side chains, are important secondary metabolites that are predominantly found in *Brassica* crops [58].

The cancer-preventive potential of kale (*B. carinata*) has been demonstrated through in vitro studies which indicated the protection of human liver cells against aflatoxin in vitro [59]. Rose et al. [60] obtained similar results with broccoli (*Brassica oleracea* var. *italica*) and watercress (*Nasturtium officinale*). Isothiocyanates—hydrolysis products of GSLs—extracted from broccoli and watercress sprouts suppressed human MDA-MB-231 breast cancer cells in vitro. In addition, extracts of 3-day-old broccoli sprouts were highly effective in reducing the incidence, multiplicity, and rate of development of mammary tumors in rats treated with the carcinogen DMBA (7,12-dimethylbenz[*a*]anthracene) [61]. Therefore, diets high in *Brassica* vegetables may contribute to the suppression of carcinogenesis, and this effect is at least partly related to their relatively high content of GSLs [62].

Among five of the microgreen species of the Brassicaceae, namely broccoli (*Brassica oleracea* var. *italica*), daikon (*Raphanus raphanistrum* subsp. *sativus*), mustard (*Brassica juncea*), rocket salad (*Eruca vesicaria*), and watercress (*Nasturtium officinale*), broccoli had the highest polyphenol, carotenoid, and chlorophyll contents, as well as strong antioxidant power [53]. Mustard microgreens showed high ascorbic acid and total sugar contents. On the other hand, rocket salad exhibited the lowest antioxidant content and activity among the five evaluated microgreen crops [53].

Broccoli, curly kale, red mustard, and radish microgreens are good sources of minerals. They provide considerable amounts of vitamin C (31–56 mg/100 g fresh weight) and total carotenoids (162–224 mg β-carotene/100 g dry weight), the latter being higher than in adult plants [63]. In digestion studies, total soluble polyphenols and total isothiocyanates showed a bioaccessibility of 43–70% and 31–63%, respectively, while the bioaccessibility of macroelements ranged from 34–90% [63]. Among the four microgreen crops tested, radish and mustard presented the highest bioaccessibility of bioactive compounds and minerals.

### 2.2. Consumer Acceptance of Sprouts and Microgreens and Nutritional Profile of Microscale Vegetables

Six commonly grown and consumed microgreen species were tested by Michell et al. [64] for consumer acceptance, as follows: (a) Brassicaceae: arugula (*Eruca sativa*), broccoli (*Brassica oleracea* var. *italica*), and red cabbage (*B. oleracea* var. *capitata*); (b) Amaranthaceae: bull’s blood beet (*Beta vulgaris*) and red garnet amaranth (*Amaranthus tricolor*); and (c) Fabaceae: tendril pea (*Pisum sativum*). All six microgreen crops received high ratings for appearance acceptability; hence they could easily be used to enhance the visual appearance of meals if they have the appropriate sensory attributes [64]. Among the six microgreen crops evaluated, broccoli, red cabbage, and tendril pea received the highest overall acceptability score with similar trends for taste and texture.

In a similar approach, Xiao et al. [39] evaluated six microgreen species for their sensory attributes and nutritional value. The six species consisted of (i) three Brassicaceae crops: Dijon mustard—*Brassica juncea*, peppercress—*Lepidium bonariense*, and China rose radish—*Raphanus sativus*; (ii) two representatives of the Amaranthaceae family: bull’s blood beet—*Beta vulgaris* and red amaranth—*Amaranthus tricolor*; and (iii) one representative of the Lamiaceae family: opal basil—*Ocimum basilicum*. Overall, all six microgreen species included in the study received “good” to “excellent” consumer acceptance ratings and showed high nutritional quality. Among those six crops, bull’s blood beet received the highest acceptability score regarding flavor and overall eating quality, while peppercress received the lowest score [39]. In addition, the authors detected the highest concentrations of total ascorbic acid and tocopherols in China rose radish, the highest contents of total phenolics and phylloquinone (vitamin K1) in opal basil, and the highest content of carotenoids in red amaranth.

In trials conducted at the World Vegetable Center, the consumer acceptance of amaranth (*Amaranthus tricolor*) landraces, conserved in the Genebank, were compared with commercially available cultivars [45]. A Genebank accession (VI044470) consistently received the highest ratings for appearance, texture, taste, and general acceptability at the sprout, microgreen, and fully grown stages.

A consumer acceptance study conducted in India comprised the following ten microgreens: carrot, fenugreek, mustard, onion, radish, red roselle, spinach, sunflower, fennel, and French basil [65]. The organoleptic acceptability of all ten microscale vegetables ranged from very good to excellent.

The high appreciation of microgreens compared to mature vegetables might also be related to their aroma profile. Recent research undertaken by Dimita et al. [66] has shown that the aroma profile of *Perilla frutescens* var. *frutescens* (Chinese basil or perilla; green leaves) and *P. frutescens* var. *crispa* (red leaves) is much higher at the microgreens stage than at the later adult stage. Both varieties have a clearly distinct aroma profile at the microscreen stage. The red variety emitted a citrusy, spicy, and woody aroma, while the green type produced a fruity, sweet, spicy, and herbaceous aroma at the microgreens stage [66]. After the microscreen stage, at the age of four weeks, green Chinese basil no longer emitted any aroma volatiles. Hence, the aroma profile of Chinese basil leaves at the microgreen stage is clearly variety-specific and not related to the content of total phenols or the antioxidant capacity of the leaves.

Attempting a nutritional determination among five Brassicaceae microgreen crops (broccoli, daikon, mustard, rocket salad, and watercress), broccoli excelled [53]. Broccoli microgreens had the highest content of isothiocyanates, known for their cancer-preventing abilities [61,62] and displayed the most potent antioxidant power. Broccoli microgreens exhibited the overall best nutritional profile and, therefore, are considered as one of the most promising functional food species [53].

Based on the determination of the contents of 11 nutrients and vitamins, as well as the anti-nutrient oxalic acid, and their relative contribution to the diet as per the estimated daily intake published in the United States Department of Agriculture (USDA) database for green leafy vegetables, Ghoora et al. [67] computed a nutrient quality score (NQS) to assess the nutritional quality of ten culinary microgreen species. The selected species included vegetable crops (spinach, carrot, mustard, radish, roselle, and onion); leguminous crops (fenugreek); oleaginous crops (sunflower); and aromatic species (French basil and fennel). All microgreen crops are moderate to good sources of protein, dietary fiber, and essential nutrients. Concerning their vitamin content, the studied microgreens are excellent sources of ascorbic acid, vitamin E, and beta-carotene (pro-vitamin A), meeting 28–116%, 28–332%, and 24–72% of reference daily intake of the respective vitamins [67]. In general, microgreens had low levels of oxalic acid, which is a predominant anti-nutrient in mature leafy vegetables. Based on the calculated NQS, radish microgreens showed the highest nutrient density, followed by French basil and roselle microgreens. On the other hand, fenugreek and onion microgreens are the least nutrient dense. Furthermore, the calculated NQS revealed that all microgreens were 2–3.5 times more nutrient dense than mature leaves of spinach cultivated under similar conditions.

While high nutrient density and high phytochemical content are considered a must in sprouts and microgreens, these microscale vegetables must also have high consumer acceptability in flavor attributes and visual appearance. Based on organoleptic and nutritional properties, Caracciolo et al. [68] assessed different microgreens species regarding consumer acceptance of appearance, texture, and flavor. The 12 microgreen species included in the studies were amaranth, coriander, cress, green basil, komatsuna, mibuna, mizuna, pak choi, purple basil, purslane, Swiss chard, and tatsoi. The results revealed that while the visual appearance of the microgreens played a role, the flavor and texture of microgreens were the main determining factors for consumer acceptance. In general, low astringency, sourness, and bitterness enhanced the consumer acceptability of microgreens [68]. Among the 12 examined microgreen species, mibuna (*Brassica rapa* subsp. *nipposinica*) and cress (*Lepidium sativum*) received the lowest consumer acceptance score, while Swiss chard (*Beta vulgaris* subsp. *vulgaris*) and coriander (*Coriandrum sativum*) were the most appreciated microscale vegetables.

Unfortunately, phenolic content strongly correlates with flavor attributes such as sourness, astringency, and bitterness. Therefore, microscale vegetables rich in phenolics, such as red cabbage (*Brassica oleracea* var. *capitata*), sorrel (*Rumex acetosa*), and peppercress (*Lepidium bonariense*), in general, receive a low consumer acceptability score [22,39]. However, rich content in minerals, vitamins, phenolics, and antioxidant activity can also be found in species of more acceptable tastes, such as amaranth, coriander, and Swiss chard [22,25,37,45,69,70]. As shown with the above examples, identifying microgreen species that satisfy both sensory and health attributes at a high degree remains a challenge since acrid taste’s acceptability is subject to both inherited and acquired taste factors [22]. Providing concise, crop-specific information about the culinary uses and the outstanding nutritional and health benefits of microscale vegetables might increase consumer interest. Such information might convince them to try products of high nutritional value but less agreeable tastes, eventually broadening the overall consumer acceptability of such produce [68].

## 3. Underutilized Species with Potential for Microscale Vegetable Production to Enhance Nutrition Security

Breeding for high yield, appearance, etc., may sometimes unintentionally lead to a decline in essential nutrients and phytochemicals. This hypothesis is supported by a review study conducted on 43 garden crops that revealed a statistically reliable reduction in six nutritional factors (protein, Ca, P, Fe, riboflavin, and ascorbic acid) between 1950 and 1999 based on USDA food composition data for this period [71]. These changes can be explained by changes in the crop varieties cultivated during this same period. Similar trends have been observed in wheat grain [72,73] and potato tubers [74]. Marles [75] conceded that some modern varieties of vegetables and grains might have lower contents in some nutrients than older varieties due to a dilution effect of increased yield by the accumulation of carbohydrates without a proportional increase in certain other nutrients. Nevertheless, he argued that eating the WHO-recommended daily servings of fruits and vegetables would provide adequate nutrition [75]. Nonetheless, we know that most countries and the majority of the global population, especially in sub-Saharan Africa, are still well below the WHO-recommended daily intake levels of fruits and vegetables [17,18].

When aiming for high phytonutrient density and exciting flavors and tastes, it might well be worth exploring farmers’ landraces, wild food plants, or populations found in a semi-wild or wild state, such as crops’ wild relatives. Such species are often part of the conservation focus of national genebanks, e.g., the genebanks maintained by the USDA in the USA, or international ones, e.g., the Genebank maintained by the World Vegetable Center (WorldVeg). This idea of exploring landraces, wild food plants, or crops’ wild relatives for microscale vegetable production has recently gained impetus [22,26,33,76,77].

The microgreens of wild plants and culinary herbs could constitute a source of functional food with attractive aromas, textures, and visual appeal, which could provide health benefits due to their elevated nutraceutical value and could be exploited in new gastronomic trends [25,38,39]. Studies of 13 wild edible plants from 11 families undertaken by Romojaro et al. [78] revealed that their outstanding nutritional value would merit promotion to provide health benefits. Fennel, which is commonly used for sprout and microgreen production, has higher radical scavenging activity, total phenolic, and total flavonoid contents in its wild form compared to medicinal and edible fennel [79]. Variations in the phytochemical content of wild fennel obtained from different geographical areas was also reported. For broccoli, kale, and pak choi, there is a variation of the concentrations of secondary plant metabolites among cultivars with ranges up to 10-fold (Table 2) [49].

Studies involving three wild leafy species, *Sanguisorba minor* (salad burnet), *Sinapis arvensis* (wild mustard), and *Taraxacum officinale* (common dandelion), at the microgreen and baby green stages were conducted by Lenzi et al. (Table 2) [33]. The authors recognized the potential of those wild edible plants in achieving competitive yields and contributing to the dietary intake of nutritionally essential macro- and microelements, as well as bioactive compounds.

Sprouted seeds of chia (*Salvia hispanica*), golden flax, evening primrose, phacelia and fenugreek are an excellent source of health-promoting phytochemicals, especially antioxidants and minerals [80]. Germination significantly increased the total phenolic content (e.g., from 1.40 to 4.54 mg GAE g^−1^ in fenugreek and from 0.33 to 5.88 mg GAE g^−1^ in phacelia), antioxidant activity (e.g., 1.5 to 52.5-fold in fenugreek and phacelia, respectively), and the content of phenolic acids and flavonoids in sprouts compared to the ungerminated seed of the mentioned species.

A rather exotic medicinal vegetable with a mild, bitter flavor is Korean ginseng (*Panax ginseng*). Sprouts of this crop can be grown to whole plants in 20 to 25 days in a soil-less cultivation system [81,82]. Their main bioactive compounds are ginsenosides which have anti-cancer, anti-diabetic, immunomodulatory, neuroprotective, radioprotective, anti-amnestic, and anti-stress properties (see references in [81]). Korean ginseng sprouts can be included in salads, milkshakes, sushi, soups, and tea. It is also used in health food supplements and cosmetics.

In summary, underutilized plants, such as farmers’ landraces, wild food plants, or crops’ wild relatives, often conserved in genebanks, might offer valuable opportunities to produce sprouts and microgreens with high nutritional value and exciting flavors and tastes, thus meeting the demands of health-conscious consumers. However, additional research efforts are required to determine whether the germination performance of these novel plant materials is satisfactory for commercial microscale vegetable production.

**Table 2:** Underutilized plant material for sprouting and microgreen production, consumer acceptance, and highlights of secondary metabolites.

Family	Species	Type of Plant Material	Secondary Metabolites	References
Amaranthaceae	*Amaranthus caudatus* (foxtail amaranth)	old varieties	High total phenolics, total betalain, and total flavonoid content	[83]
	*Amaranthus cruentus* (red amaranth)	old varieties	Amaranth sprouts are a good source of anthocyanins and total phenolics with high antioxidant activity	[83,84]
	*Amaranthus hypochondriacus* (Prince’s feather)	ornamental	Good source of antioxidants, especially the leaves	[83]
	*Amaranthus tricolor* (edible amaranth)	landrace	A genebank accession (VI044470) consistently received the highest ratings for appearance, texture, taste, and general acceptability at the sprout, microgreen, and fully grown stage compared to commercial cultivars	[45]
	*Atriplex hortensis* (red orach)	under-utilized	Ascorbic acid content	[69]
	*Chenopodium album* (pigweed)	under-utilized	Antioxidant activity and total phenolic content are enhanced in germinated *C. album* seeds	[85]
	*Chenopodium quinoa* (quinoa)	old variety	Quinoa sprouts are a good source of anthocyanins and total phenolics with high antioxidant activity	[84]
Apiaceae	*Anethum graveolens* (dill)	under-utilized	Total phenolic and total flavonoid content; antioxidant activity	[86]
	*Coriandrum sativum* (coriander)	under-utilized	A strong influence of the substrate on the content of carotenoids and total phenolics	[87]
Araliaceae	*Panax ginseng* (Korean ginseng)	under-utilized medicinal plant	Ginsenosides (triterpene glycoside saponin)	[81,82]
Asteraceae	*Artemisia dracunculus* (tarragon)	aromatic herb	N/A; red and blue LED exposure enhances germination and growth of tarragon sprouts	[88]
	*Cichorium intybus* (chicory)	medicinal herb	Total phenolics, tocopherols, anthocyanins, high levels of carotenoids	[89]
	*Taraxacum officinale* (common dandelion)	wild plants	Anthocyanins and carotenoids; high Fe content	[33]
Basellaceae	*Basella alba* (Malabar spinach)	underutilized vegetable	High ascorbic acid and total phenolic content	[90]
Boraginaceae	*Borago officinalis* (borage)	medicinal herb	Total phenolic and carotenoid content, antioxidant capacity	[91]
	*Phacelia tanacetifolia* (phacelia)	wildflower	Total phenolics, flavonoids, and antioxidant activity	[80]
Brassicaceae	*Brassica oleracea* var. *italica* (broccoli)	landrace	(1) High polyphenol content in broccoli landrace; (2) highest vitamin C content found in microgreens of broccoli landrace	[76]
	*Brassica oleracea* var. *acephala* (kale)	landrace	(1) Higher content of flavonoids (quercetin and kaempferol derivatives) in traditional cultivars than in modern cultivars (hybrids); (2) among 8 cultivars, higher concentrations of lutein and β-carotene were found in old cultivars	[49]
	*Sinapis arvensis* (field mustard)	under-utilized	Carotenoids and anthocyanins	[33]
	*Wasabi japonica* (wasabi)	under-utilized	Ascorbic acid, β-carotene, lutein/zeaxanthin content	[69]
Convolvulaceae	*Ipomoea aquatica* (water spinach)	under-utilized	High total phenolics and total flavonoid content; high antioxidant activity	[89,90]
Cucurbitaceae	*Cucumis sativus* (cucumber)	under-utilized	High ascorbic acid content	[90]
	*Cucurbita moschata* pumpkin)	under-utilized	High total phenolics and total flavonoids content	[90]
	*Lagenaria siceraria* (bottle gourd)	under-utilized	High total phenolics content; high antioxidant activity; high Cu and Fe levels	[90]
Fabaceae	*Glycine max* (soybean)	landrace	Nutrient and antioxidant contents of soybean sprouts were superior to mungbean sprouts	[92]
	*Medicago intertexta* (hedgehog medick)	wild species	Total phenolic and flavonoid contents, antioxidant, and antidiabetic activities	[93]
	*Medicago polymorpha* (bur clover)	wild, invasive species	Total phenolic and flavonoid contents, antioxidant, and antidiabetic activities	[93]
	*Melilotus indicus* (annual yellow sweet clover)	wild species	Total phenolic and flavonoid contents, antioxidant, and antidiabetic activities	[93]
	*Vigna radiata* (mungbean)	landrace	(1) Old mungbean accessions were superior in protein, calcium (Ca), iron (Fe), zinc (Zn), carotenoid, and vitamin C content compared to improved mungbean lines at the fully mature stage; (2) compared to commercial mungbean varieties, a landrace from Taiwan (VI000323) showed the highest levels of caffeic acid and kaempferol at the sprouting and fully mature stage	[92]
Lamiaceae	*Ocimum basilicum* (Sweet basil)	culinary herb	High phylloquinone and total phenolics concentration	[39]
	*Ocimum x africanum* (lemon basil)	culinary herb	Total phenolic and total flavonoid content; antioxidant activity	[86]
	*Ocimum sanctum* (sacred basil)	medicinal herb	Total phenolic and total flavonoid content; antioxidant activity	[86]
	*Salvia hispanica* (chia)	under-utilized	Total phenolics, flavonoids, antioxidant activity.	[80]
Linaceae	*Linum flavum* (golden flax)	under-utilized	Total phenolics, flavonoids, antioxidant activity.	[80]
Malvaceae	*Corchorus olitorius* (jute mallow)	under-utilized	High ascorbic acid and total phenolics content; high antioxidant activity	[90]
	*Hibiscus subdariffa* (red roselle)	under-utilized culinary herb	Anthocyanins, flavonoids, and phenolic acids contribute to the antioxidative activity	[65]
Onagraceae	*Oenothera biennis* (evening primrose)	under-utilized	Total phenolics, flavonoids, antioxidant activity	[80]
Plantaginaceae	*Plantago coronopus* (buck’s-horn plantain)	wild herb	Total phenolics, flavonoids, and antioxidant activity	[94]
Polygonaceae	*Rumex acetosa* (sorrel)	wild herb	Total phenolics, flavonoids, and antioxidant activity	[94]
Portulacaceae	*Portulaca oleracea* (purslane)	wild herb	Total phenolics, flavonoids, and antioxidant activity	[94]
Rosaceae	*Sanguisorba minor* (salad burnet)	under-utilized	Carotenoids and anthocyanins; high amounts of Mg, P, Zn, Mn, and Mo	[33]

## 4. Variation of Nutritional Value and Content of Phytochemicals According to Plant Growth Stages

**Figure 4:**
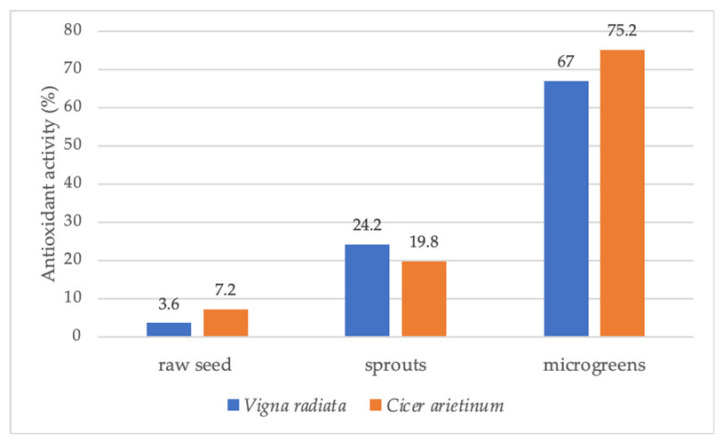
The antioxidant activity (%) in methanol extract (100 mg/mL) of raw seed, sprouts, and microgreens of *Vigna radiata* and *Cicer arietinum*; a graphical representation of data published by Kurian and Megha [42].

**Figure 5:**
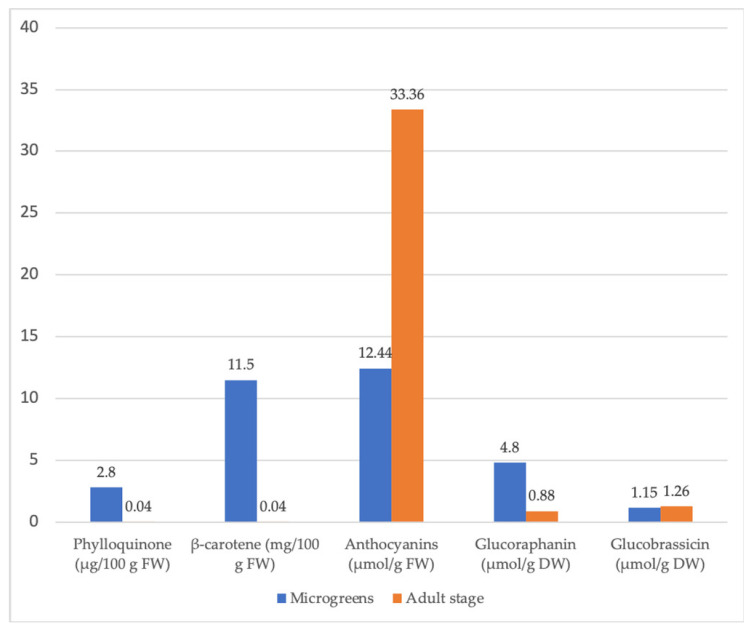
A comparison of selected phytochemical concentrations of red cabbage (*Brassica oleracea* var. *capitata*) at the microgreen and adult growth stage. FW = fresh weight; DW = dry weight; a graphical representation of data published by Choe et al. [95].

Numerous studies have shown that the nutritional value and content of phytochemicals of vegetables and other crops may vary with plant growth and development. The concentration of essential minerals, vitamins, bioactive compounds, and antioxidant activity often increases in this sequence: raw seeds—sprouted seeds—microgreens (Figure 4) [41]. In many cases, sprouts and microgreens even exceed the nutritional value of fully grown plants (Figure 5) [95]. Examples of variations of the content of essential nutrients, vitamins, and phytochemicals according to plant growth stages (seeds, sprouts, microgreens, baby leaves, and fully grown) are listed in Table 3 and discussed below.

Research undertaken by Pająk et al. [96] and Khang et al. [97] has shown that seed germination can increase total phenolic content (TPC) levels and antioxidant activity in mungbean (*Vigna radiata*), adzuki bean (*V. angularis*), black bean (*V. cylindrica*), soybean (*Glycine max*), peanut (*Arachis hypogaea*), radish (*Raphanus sativus*), broccoli (*Brassica oleracea* var. *italica*), and sunflower (*Helianthus annuus*). Among the 13 phenolic compounds detected in high concentrations in the studies carried out by Khang et al. [97], sinapic acid, ellagic acid, ferulic acid, and cinnamic acid showed high correlations with antioxidant activities. High TPC levels have also been confirmed in sprouted seeds of several underutilized species, such as chia (*Salvia hispanica*), golden flax (*Linum flavum*), phacelia (*Phacelia tanacetifolifa*), fenugreek (*Trigonella foenum-graecum*), and evening primrose (*Oenothera biennis*) [80]. Evening primrose showed the highest TPC values and antioxidant activity among those underutilized species, both for sprouts and seeds. Compared to dry grains, seed sprouting enhanced TPC levels of chia from 0.92 to 4.40 mg gallic acid equivalent (GAE) g^−1^, golden flax from 0.93 to 4.50 mg GAE g^−1^, phacelia from 0.33 to 5.88 mg GAE g^−1^, and fenugreek from 1.40 to 4.64 mg GAE g^−1^ [80].

Compared to ungerminated seeds, amaranth and quinoa sprouts showed higher contents of total flavonoids, phenolics, and antioxidant activity (Table 3) [83,84,98,99]. A substantial increase in vitamin C (ascorbic acid) content was observed from amaranth sprouts to microgreens (2.7-fold) and from amaranth microgreens to fully grown leafy amaranth (2.9-fold) (Table 3) [45]. Higher ascorbic acid and α-tocopherol levels were detected in spinach microgreens compared to the mature vegetable stage [67]. The often-higher ascorbic acid (vitamin C) content of microgreens compared to sprouts [25,92] can be explained by the presence of photosynthetic activity, which is absent in sprouts. Ascorbate is synthesized from photosynthetic hexose products and plays a significant role in photosynthesis as an enzyme cofactor (including in the synthesis of plant hormones and anthocyanins) and cell growth regulation [100]. Red cabbage microgreens had a 6-fold higher concentration of total ascorbic acids than mature cabbage [69]. With 131.6 mg/100 g FW, garnet amaranth also had a much higher total ascorbic acid (TAA) concentration than its mature counterpart [69]. With some exceptions (e.g., golden pea tendrils and sorrel), most of the 25 microgreens studied by Xiao et al. [69] showed higher TAA concentration than their mature counterparts.

The ascorbic acid content of fenugreek, spinach, and roselle microgreens reached 120%, 127%, and 119%, respectively, of their fully grown, mature stage (Table 3) [67]. The ascorbic acid levels of the studied microgreens ranged from 29.9–123.2 mg/100 g, and, therefore, were comparable to those of citrus fruits, which are generally known to be rich sources of vitamin C [101].

Tocopherols and tocotrienols belong to the vitamin E family. The α-tocopherol levels of fenugreek, spinach, and roselle microgreens were significantly higher than those of their respective mature leaves (Table 3) [67]. Among the 25 microgreens evaluated, green daikon radish microgreens exhibited the highest tocopherol concentrations in the α- and γ-forms, followed by coriander, opal radish, and peppercress [69]. Although golden pea tendrils had the lowest tocopherol concentrations of α (4.9 mg/100 g FW) and γ (3.0 mg/100 g FW), these values were still higher than those determined in mature spinach leaves.

In general, the microgreens’ phylloquinone (vitamin K1) content is relatively high compared to the corresponding values of mature vegetables (Table 3) [95]. A total of 18 out of 25 commercially grown microgreens contain similar or greater phylloquinone concentrations than the commonly consumed vegetable broccoli. Green (pea tendrils) or bright red (garnet amaranth) microgreens often exhibit higher phylloquinone concentrations than yellow microgreens (popcorn and golden pea) [69].

Among 25 microgreens, Choe et al. [95] detected wide ranges of β-carotene concentrations. Red sorrel exhibited the highest β-carotene concentration (12.1 mg/100 g FW), while golden pea tendrils and popcorn microgreens had the lowest β-carotene concentrations (0.6 mg/100 g FW). With 11.7 mg/100 g FW, coriander microgreens had the second highest β-carotene concentration, a 3-fold higher concentration than found in mature coriander leaves. With 11.5 mg/100 g FW, red cabbage microgreens had a 260-fold higher β-carotene content than that found in mature red cabbage (0.044 mg/100 g FW) [95]. Except for golden pea tendrils and popcorn shoots, most microgreens were rich in β-carotene. Coriander (10.1 mg/100 g FW) and red cabbage (8.6 mg/100 g FW) microgreens had 11.2- and 28.6-fold higher lutein/zeaxanthin concentrations, respectively, than their mature crops [69]. Coriander microgreens also exhibited the highest violaxanthin concentration.

Microscale *Brassica* vegetables (sprouts, microgreens, and baby leaves) of broccoli, kale, and radish are good sources of health-promoting phytochemicals with high antioxidant capacities. These are, in general, found in higher concentrations at the sprout and microgreen stage than in the respective adult edible plant organs (Table 3) [76,102]. In the studies undertaken by Di Bella et al. [76], polyphenol profiles differed among the three novel food types (sprouts, microgreens, and baby leaves) and cultivars within the same food type. Sprouts showed the highest total polyphenol content of the broccoli cultivars and the highest antioxidant capacity of all three cultivars studied (Table 3) [76]. Ascorbic acid levels varied significantly among the studied cultivars and the three plant growth stages. Microgreens of the landrace ‘Broccolo Nero’ presented the highest ascorbic acid values [76]. Chicory, lettuce, and broccoli microgreens showed higher amounts of α-tocopherol and carotenoids than mature vegetables (Table 3) [89]. Health-promoting phytochemicals are more concentrated in cruciferous sprouts (e.g., broccoli and red radish) than in their respective adult plant edible organs.

In the Fabaceae vegetables chickpea and mungbean, the content of total phenolics and vitamins and the antioxidant activity increased in the sequence of raw seeds, sprouts, and microgreens [42]. The sprouting of mungbean seeds increased total phenolic and flavonoid (TF) levels and the antioxidant activity (AA) when compared to ungerminated seeds (Table 3) [96]. Compared to sprouts, flaxseed (*Linum usitatissimum*) microgreens exhibited higher chlorophyll, carotenoid, and phenol contents, as well as higher antioxidant capacity [103].

Yadav et al. [90] studied nine leafy summer vegetables’ mineral content and antioxidant activity at the microgreen and mature stages. While microgreens had a higher content of K and Zn, no specific trend was observed for Cu, Fe, and Mn [90]. Microgreens of jute (*Corchorus olitorius*) and cucumber (*Cucumis sativus*) presented higher ascorbic acid levels (34.9 mg/100 g fresh weight (FW) and 25.0 mg/100 g FW, respectively) as compared to their mature stages (10.0 mg/100 g FW and 17.45 mg/100 g FW, respectively) [90]. The ascorbic acid content of water spinach (*Ipomoea aquatica*) was comparable at the microgreen and mature stages. For other vegetable species, including bottle gourd (*Lagenaria siceraria*), pumpkin (*Cucurbita moschata*), amaranth (*Amaranthus tricolor*), Malabar spinach (*Basella alba*), radish (*Raphanus raphanistrum*), and beet (*Beta vulgaris* var. *bengalensis*), the mature plants showed higher ascorbic acid contents in comparison with the microgreen stage.

Although the ascorbic acid content is often higher at the adult stage than the microgreen stage, the human body cannot appropriately benefit from this rich ascorbic acid source. Leafy vegetables at the mature stage are generally consumed after cooking, and ascorbic acid is known to be thermolabile [56]. In contrast, microgreens are usually consumed fresh; hence, the human body can fully benefit from this ascorbic acid source in microgreens. Jute (*Corchorus olitorius*) and water spinach (*Ipomoea aquatica*) are richer sources of phenolics and flavonoids compared to commonly consumed vegetable crops such as broccoli, lettuce, and carrot at the mature stage [90].

Weber [104] studied the mineral content of lettuce (*Lactuca sativa*) and cabbage (*Brassica oleracea* var. *capitata*) microgreens and compared them to their mature vegetable stage. The average ratios across ten nutrients (P, K, Ca, Mg, S, Mn, Cu, Zn, Na, and Fe) indicated that hydroponically grown lettuce and cabbage microgreens were 2.7 and 2.9 times, respectively, more nutrient-rich than their corresponding mature vegetables (Table 3). When microgreens were cultivated on vermicompost, their nutritional superiority over the adult stage was even more pronounced. In a similar experiment with broccoli microgreens, eight minerals were analyzed that are commonly reported in nutrition information facts for foods (P, K, Ca, Mg, Mn, Fe, Zn, and Na) [105]. In these studies, the average nutrient ratio of vermicompost-grown broccoli microgreens to fully grown broccoli was 1.73 [105]. Based on this experimentally verified ratio, Weber [105] argued that one would need to eat ca. 42% less mass of microgreens (ca. 53 g FW) to obtain the same amount of minerals present in a serving of raw broccoli florets (91 g). Furthermore, broccoli microgreens would require 158–236 times less water to grow than a nutritionally equivalent amount of broccoli vegetable in fields in California’s Central Valley [105].

Pinto et al. [106] reported a high nutritional content of 2-week-old butterhead lettuce (*Lactuca sativa* var. *capitata*) microgreens. The content of essential minerals such as Ca, Mg, Fe, Mn, Zn, Se, and Mo was higher in lettuce microgreens than in mature lettuces. High nitrate levels (NO_3_^−^) may accumulate in leafy vegetable crops (e.g., cabbage, spinach, and lettuce), and breeders aim to breed leafy vegetables with low nitrate contents. Nitrate that remains unassimilated in vegetable plant tissues can be enzymatically converted to the more toxic nitrite (NO_2_^−^) during storage and food processing. NO_3_^−^ ingested by humans can also be reduced to NO_2_^−^ through the activity of gut microorganisms [107]. Nitrite is a potent carcinogen and may cause the accumulation of methemoglobin, a compound with potentially toxic effects on human health [108], particularly in infants and children. Therefore, it is essential to note that lettuce microgreens have a much lower NO_3_^−^ content than mature lettuces and are thus safe for consumption by infants and children [106]. A lower concentration of nitrates in Swiss chard (*Beta vulgaris* subsp. *vulgaris*) and rocket (*Eruca sativa*) microgreens than typically found in the corresponding baby leaf or adult vegetables was reported by Bulgari et al. [109]. Withholding nutrient supplementation in the growing media of microgreens is another option almost suppressing nitrate accumulation completely. The production of brussels sprouts (*Brassica oleracea* var. *gemmifera*) and cabbage (*B. oleracea* var. *capitata*) microgreens without nutrient supplementation led to a 99% decrease in the nitrate content while maintaining steady calorimetric qualities and total phenolic acid contents with only minor yield reduction [110]. Under nutrient deprivation, cabbage microgreens even showed a 30% increase in total ascorbic acid and a 12% increase in total anthocyanins.

Protection against carcinogenesis, mutagenesis, and other forms of toxicity can be achieved by the induction of phase 2 detoxication enzymes [61]. Large quantities of inducers of enzymes that protect against carcinogens can be delivered through dietary means by small amounts of young crucifer sprouts. For example, three-day-old broccoli sprouts contained as much inducer activity as 10–100 times larger quantities of the corresponding mature vegetable [61]. This is a tremendous health benefit of the *Brassica* microscale vegetables which are easily accessible to consumers.

In addition to *Brassica* microscale vegetables, okra (*Abelmoschus esculentus*) and water spinach (*Ipomoea aquatica*) sprout extracts also exhibited anti-proliferative effects on gastric cancer, hepatoma, and melanoma cell lines [111]. However, alfalfa and pea sprout extracts showed negligible anti-cancer activity. Matsuo et al. [111] hypothesized that the water-soluble bioactive compounds in okra and water spinach sprouts are responsible for the observed anti-cancer activities.

Besides macro- and microelements, vitamins, polyphenols, and other bioactive compounds, dietary fiber (DF) is another essential component of the human diet. The macromolecules of DF mainly consist of plant cell wall components, polysaccharides, and lignin. They resist digestion by endogenous enzymes in the human gut and promote the satiety sensation [112]. The health benefits of DF include weight loss, prevention and treatment of constipation, control of serum cholesterol levels, and improved glucose tolerance, among others [67,112]. In addition, the ability of DF to bind toxic compounds has been recognized as a protective mechanism against cancer.

The studies conducted by Paradiso et al. [89] indicated relatively low DF contents ranging from 0.3 to 0.7 g per 100 g FW in broccoli and chicory microgreens, respectively. In contrast, Ghoora et al. [67] reported total dietary fiber (TDF) contents ranging from 1.41 g/100 g FW in French basil and 3.63 g/100 g FW in sunflower to 4.28 g/100 g FW in fennel. Radish and roselle microgreens showed relatively high values of soluble dietary fiber of 0.28 and 0.29 g/100 g FW, respectively. Thus, the TDF values of sunflower and fennel microgreens are in the same range as mature leafy vegetables, known for their high TDF contents ranging from 3.0 g/100 g FW in cabbage to 4.9 g/100 g FW in fenugreek leaves [112]. It is obvious that with the increasing age of microgreens, their TDF content is expected to increase as well.

The examples in this section indicate that microscale vegetables are, in general, nutrient-dense and rich in phytochemicals, often with a reduced level of antinutrients as compared to the adult growth stage, hence constituting an attractive component as a functional food in the diet of health-conscious consumers.

**Table 3:** The variations in contents of nutrients and phytochemicals according to plant growth stages (seeds, sprouts, microgreens, baby leaves, and fully grown).

Family	Species	Secondary Metabolites	Reference
Amaranthaceae	*Amaranthus caudatus* (foxtail amaranth)	Amaranth sprouts showed significantly higher contents of total flavonoids, rutin, amaranthine, and iso-amaranthine than ungerminated seeds.	[83]
	*Amaranthus cruentus* (red amaranth)	Amaranth sprouts have a significantly higher antioxidant activity than seeds, which may be a result of the difference in the content of polyphenols, anthocyanins, and other compounds.	[84]
	*Amaranthus tricolor* (edible amaranth)	(1) Mean protein, Fe and Zn content were considerably higher in amaranth sprouts compared with amaranth microgreens; (2) a substantial increase in vitamin C content from amaranth sprouts to microgreens (2.7-fold) and from amaranth microgreens to fully grown leafy amaranth (2.9-fold); (3) α-carotene and β-carotene were detected in all three growth stages and content increased considerably from sprouts to microgreens.	[45]
	*Chenopodium quinoa* (quinoa)	Quinoa sprouts have a significantly higher antioxidant activity than seeds.	[84]
	*Chenopodium quinoa*	Total phenol content and antioxidant activity increase with the sprouting of seeds.	[98]
	*Chenopodium quinoa*	Sprouts have significantly higher antioxidant capacity values after four days of germination than raw seeds; (2) phenolic content values of 4-day-old sprouts are about 2.6 times higher than seeds.	[99]
	*Spinacia oleracea* (spinach)	Higher ascorbic acid and α-tocopherol levels in microgreens compared to the mature stage.	[67]
Asteraceae	*Helianthus annuus* (sunflower)	Sprouting increased total phenolic and flavonoid levels, as well as the antioxidant activity compared to ungerminated seeds.	[96]
	*Lactuca sativa* (lettuce)	Sprouts showed higher amounts of α-tocopherol and carotenoids compared to mature lettuce.	[89]
	*Lactuca sativa*	The average ratio of ten nutrients (P, K, Ca, Mg, S, Mn, Cu, Zn, Na, and Fe) indicated that hydroponically grown lettuce microgreens were 2.7 times more nutrient-rich than mature lettuce.	[104]
	*Lactuca sativa* var. *capitata* (butterhead lettuce)	The content of essential minerals such as Ca, Mg, Fe, Mn, Zn, Se, and Mo was higher and nitrate content was lower in lettuce microgreens than in mature lettuces.	[106]
Boraginaceae	*Phacelia tanacetifolia* (phacelia)	TPC and antioxidant activity were higher in sprouts than in ungerminated seeds.	[80]
Brassicaceae	*Brassica oleracea* var. *capitata* (cabbage)	The average ratio of ten nutrients (P, K, Ca, Mg, S, Mn, Cu, Zn, Na, and Fe) indicated that hydroponically grown cabbage microgreens were 2.9 times more nutrient-rich than mature cabbage.	[104]
	*Brassica oleracea* var. *capitata*	Higher total ascorbic acid, phylloquinone, β-carotene, and glucoraphanin in cabbage microgreens than in mature cabbage.	[95]
	*Brassica oleracea* var. *italica* (broccoli)	(1) Sprouts showed significantly higher polyphenol values than microgreens and baby leaves; (2) high increments of kaempferol and apigenin in broccoli landrace from the seed to the baby leaves growth stage; (3) antioxidant levels were highest in sprouts and tended to decrease with further growth.	[76]
	*Brassica oleracea* var. *italica*	Sprouting increased total phenolic and flavonoid levels, as well as the antioxidant activity compared to ungerminated seeds.	[96]
	*Brassica oleracea* var. *italica*	Health-promoting phytochemicals are more concentrated in cruciferous sprouts (e.g., broccoli and red radish) than in the adult plant edible organs.	[102]
	*Brassica oleracea* var. *italica*	3-day-old broccoli sprouts contained a much higher inducer activity of detoxication enzymes than the corresponding mature vegetable.	[61]
	*Brassica oleracea* var. *italica*	Broccoli sprouts showed higher amounts of α-tocopherol and carotenoids compared to mature broccoli.	[89]
	*Brassica oleracea* var. *italica*	10-fold higher content of glucobrassicin in broccoli microgreens compared to the mature stage.	[95]
	*Brassica oleracea* var. *acephala* (kale)	Sprouts showed significantly higher polyphenol values than microgreens and baby leaves.	[76]
	*Brassica rapa* subsp. *chinensis* (pak choi)	Decreasing content of 3-butenyl glucosinolates from sprouts to adult leaves.	[49]
	*Cichorium intybus* (chicory)	Sprouts showed higher amounts of α-tocopherol and carotenoids compared to mature chicory.	[89]
	*Eruca sativa* (arugula)	Higher content of total ascorbic acid, phylloquinone, and β-carotene in arugula sprouts compared to the mature stage.	[95]
	*Raphanus sativus* (radish)	Health-promoting phytochemicals are more concentrated in cruciferous sprouts (e.g., broccoli and red radish) than in the respective adult plant edible organs.	[102]
	*Raphanus sativus*	Sprouting increased total phenolic and flavonoid levels and the antioxidant activity compared to ungerminated seeds; radish (and sunflower) sprouts were the richest in phenolic compounds.	[96]
Fabaceae	*Cicer arietinum* (chickpea)	Chickpea microgreens contained higher vitamins and higher antioxidant activity than raw seeds and sprouts.	[42]
	*Trigonella foenum-graecum* (fenugreek)	Higher ascorbic acid and α-tocopherol levels in microgreens compared to the mature stage.	[67]
	*Vigna radiata* (mungbean)	Sprouting mungbean seeds enhanced vitamin C content 2.7-fold compared to mature mungbean grain.	[92]
	*Vigna radiata*	Mungbean sprouts showed increased total phenolic (TPC) and total flavonoid (TF) contents and higher antioxidant activity (AA) than ungerminated seeds; radish and sunflower sprouts were superior to mungbean sprouts regarding TPC, TF, and AA levels.	[96]
	*Vigna radiata*	The total phenolics and vitamins content increased in the sequence of raw seeds, sprouts, and microgreens.	[42]
	*Glycine max* (soybean)	(1) Isoflavones were found at high concentrations in soybean sprouts and could easily provide the recommended anticarcinogenic dose range from 1.5 to 2.0 mg/kg of body weight per day; (2) The vegetable soybean stage was nutritionally superior to soybean sprouts in terms of the content of protein (14% increase), Zn (45%), Ca (72%), and Fe (151%).	[92]
Linaceae	*Linum usitatissimum* (flaxseed)	Microgreens exhibited a higher chlorophyll (+62.6%), carotenoid (+24.4%), and phenol content (+37.8%), as well as higher antioxidant capacity (+25.1%) than sprouts.	[103]
Malvaceae	*Hibiscus sabdariffa* (roselle)	Higher ascorbic acid and α-tocopherol levels in microgreens compared to the mature stage.	[67]

## 5. Environmental and Priming Factors That Have an Impact on the Nutrient and Phytochemical Content of Sprouts and Microgreens

As shown in previous sections of this paper, many factors determine the contents of nutrients and phytochemicals in microscale vegetables, such as the selected crop and cultivar, the chosen genotype’s breeding status, and the growth stage. Other factors that may impact the nutritional quality of microscale vegetables are the environment in which they are grown, the selected illumination, substrates used, nutrient biofortification, and salinity stress. On the other hand, packaging methods and storage temperature help retain nutrients and phytochemicals [37]. All these factors may influence microscale vegetables’ photosynthetic and metabolic activities and may improve nutritional quality, depending on the crop/species and genotype used.

### 5.1. The Effect of Growth Environment and Growing Substrates

Microgreens can be easily self-produced by consumers at home or commercially grown using controlled environment agriculture (CEA). However, recent research has shown that the cultivation environment might influence the composition of secondary metabolites, such as polyphenols and glucosinolates. This has been the case with kale and broccoli microgreens grown under commercial (growth chamber) and home-grown (windowsill) environments [52]. Windowsill-grown microgreens showed higher concentrations of hydroxycinnamic acid esters of flavanols than those produced in a growth chamber. On the other hand, the contents of 4-methoxyglucobrassicin and neoglucobrassicin were higher in microgreens grown under a controlled environment.

The substrates used for microgreen production significantly impact the nutrient content per gram of fresh weight of plant material. Cabbage microgreens grown on vermicompost had considerably higher concentrations of K, S, Ca, Mg, Mn, Cu, Zn, Fe, and Na than hydroponically grown cabbage [104]. Exceptionally high nutrient ratios for Fe were detected in cabbage microgreens grown on vermicompost (54.6-fold content of mature cabbage), while cabbage microgreens grown hydroponically still exceeded mature cabbage by a factor of 5.4. Similarly, lettuce microgreens grown on vermicompost showed significantly larger quantities of K, S, Ca, Mn, Zn, Fe, and Na than hydroponically grown lettuce microgreens [104]. Regarding Zn, cabbage microgreens had a 5 to 7.5 times higher nutrient ratio than mature cabbage. Microgreens are apparently able to absorb significant amounts of essential micronutrients from nutrient-rich food wastes that accumulate in households (mainly fruit and vegetable wastes) and become bioavailable in vermicompost.

### 5.2. Response to Environmental Stresses

Polyphenols play a fundamental role in the defense system of plants against heavy metals, salinity, drought, extreme temperatures, pesticides, and ultraviolet (UV) radiations [113]. In response to environmental stresses, plants produce diverse metabolites, which also contribute to the functional quality of edible plant parts, such as mineral nutrients, amino acids, peptides, proteins, vitamins, pigments, and other primary and secondary metabolites [114]. The application of eustress, i.e., mild to moderate salinity or nutritional stress, can elicit targeted plant responses by activating physiological and biochemical mechanisms. These, in turn, may lead to the accumulation of desired bioactive compounds in the harvested produce (see the literature review by Rouphael and Kyriacou [115]). Salinity eustress may enhance health-promoting phytochemicals such as lycopene, β-carotene, vitamin C, and polyphenols in vegetables [115]. For example, exposing Se-biofortified maize grains to mild NaCl stress (i.e., 25 mM NaCl) during germination resulted in good sprout yields, increased the content of selenocysteine, and boosted the synthesis of pro-nutritional semipolar metabolites with antioxidant properties [116].

Nutrient deprivation in wild rocket (*Diplotaxis tenuifolia*) microgreen production elicited a substantial increase in secondary metabolites, such as lutein (110%), β-carotene (30%), total ascorbic acid (58%), and total anthocyanins (20%); however, with a concomitant significant yield reduction of 47% [110]. On the other hand, moderate nutrient stress (half-strength nutrient solution-NS) applied to red Salanova butterhead lettuce (*Lactuca sativa* var. *capitata*) enhanced the concentrations of total ascorbic acid, total phenolic acids, and anthocyanins by 266%, 162%, and 380%, respectively, compared to the control, grown under full-strength NS [117]. For the above reasons, mild salinity, unbalanced mineral nutrition, or complete nutrient deprivation in the growth solution of soil-less culture systems for microscale vegetable production may prove helpful to naturally modulate the levels of functional compounds, such as ascorbate, carotenoids, and phenols. Moreover, it may also curtail anti-nutrients such as nitrate [110].

Sulfur is essential in the biosynthesis of secondary metabolites, such as glucosinolates in *Brassica* crops. Levels of sulfur and/or nitrogen nutrition during plant growth may result in significant changes in the phenolic content of edible plant parts, especially flavonoids and hydroxycinnamic acid derivatives [52]. Sulfur fertilization significantly improved the antioxidant activity of two ecotypes of spring broccoli, also known as Italian turnip (*Brassica rapa* subsp. *sylvestris* var. *esculenta*). It was associated with a genotype-dependent significant reduction in leaf nitrate content [118].

Plant stress caused by withholding irrigation during the head formation of cabbage (*Brassica oleracea* var. *capitata*) led to an increase in the concentration of bioactive glucosinolates [119]. However, this gain in nutritional value must be balanced with an eventual yield loss.

Environmental shocks such as high light (exposure to a light intensity of 700 µmol m^−2^ s^−1^ for 1 day) and chilling (exposure to 4 °C at a light intensity of 120 µmol m^−2^ s^−1^ for 1 day) enhanced the total phenolic content in sprouts of alfalfa (*Medicago sativa*), broccoli (*Brassica oleracea* var. *italica*), and radish (*Raphanus sativus*) [120]. The enhanced phenolic content was correlated with higher antioxidant activity, and dry biomass accumulation was unaffected. High light produced a more robust response than chilling in enhancing the content of individual phenolic compounds. Similarly, kale sprouts (*B. oleracea* var. *acephala*) exposed to low-temperature stress (growth temperature of 8 °C with intermittent freezing for one hour at −8 °C) increased the total content of phenolic acids and glucosinolates. However, such a treatment should be used with caution, as it also led to a significant decrease in the content of carotenoids and total flavonoids [121].

Radiation with short wavelengths, such as ultraviolet (UV) lights (200–400 nm), stimulates the production of pigments that absorb light and enhance leaf coloring, such as chlorophylls and carotenoids [122]. UV radiation may also induce physiological and metabolic stress responses in plants, such as the production of antioxidant systems, the activation of reparative enzymes, the expression of genes involved in UV protection and repair, and the accumulation of UV-absorbing compounds (e.g., phenolics and carotenoids) and defense-related (e.g., glucosinolates) phytochemicals [123]. This effect of UV light has been applied to broccoli sprouts to induce the biosynthesis and accumulation of flavonoids and glucosinolates [124]. Within 24 h after application of low UV-B (280–320 nm) doses, the flavonoids kaempferol and quercetin and glucosinolates accumulated in broccoli sprouts. A single exposure of broccoli sprouts to UV-B and UV-A (320–400 nm) for 120 min before the harvest was shown to enhance the phenolic and glucosinolate contents [125]. A synergistic effect in the accumulation of neoglucobrassicin was observed by exposing broccoli sprouts to a combination of UV irradiation and sprays of the phytohormone methyl jasmonate (25 µM). A single application of UV-B triggered the production of aliphatic or specific indole glucosinolates [125].

Exposing kale (*Brassica oleracea* var. *sabellica*) sprouts to periodical low UV-B treatments on days 3, 5, 7, and 10 of sprouting, with the four treatments reaching a total dose of either 10 or 15 kJ m^−2^, is a helpful tool to stimulate the biosynthesis of phytochemicals without compromising sprout growth [126]. During sprouting, repeated UV-B treatments increased the total phenolic content of kale sprouts by 30%, stimulating the synthesis of glucosinolates (glucoraphanin and glucobrassicin) by 30% and enhancing the antioxidant activity by 20%. Therefore, the periodic application of low UV-B doses during sprout growth can optimize the content of phytochemicals in microscale vegetables.

Postharvest exposure of broccoli and radish sprouts to abiotic stress treatment in the form of UV-B radiation enhanced total phenolic content (TPC) and total antioxidant capacity (TAC) after a shelf life of 10 days at 4 °C [127]. UV-B treatment also enhanced the glucosinolates content of both crops by about 30%, while the content of sulforaphane increased by 38% in broccoli sprouts and 72% in radish sprouts.

Zlotek et al. [128] compared the effect of thermal (2-day-old sprouts exposed for 2 h to 40 °C), osmotic (NaCl exposure), and oxidative (H_2_O_2_ exposure) stresses on adzuki bean (*Vigna angularis*) sprouts. Their research revealed that only thermal stress enhanced the antioxidant activity of extracts obtained from the adzuki bean seed coat [128]. Similarly, Świeca et al. [129] were able to demonstrate that both low (4 °C) and high (40 °C) temperature stress may cause an increase in the content of polyphenols and enhance the antioxidant properties of lentil (*Lens culinaris*) sprouts.

Randhir et al. [130] have shown that natural elicitors such as fish protein hydrolysates, lactoferrin, and oregano extract may significantly improve the phenolic, antioxidant, and antimicrobial properties of 1-day-old mungbean sprouts. Elicitation of broccoli sprouts with autoclaved cultures of *Saccharomyces cerevisiae* (brewer’s yeast) and water extracts of *Salix daphnoides* (Daphne willow) bark was also effective [131]. The most effective elicitor concentrations for the increase in the content of phenolics and enhancement of antioxidant activity of broccoli sprouts were 1% bark extract of *S. daphnoides* and 0.5% of *S. cerevisiae* cultures.

The preharvest treatment of broccoli microgreens with 10 mM calcium chloride (CaCl_2_) to extend their shelf life led to a significant increase in aliphatic and indolic glucosinolates [132]. The raised glucosinolate (GLS) levels may have been responsible for the strengthened stress tolerance and defense mechanisms of broccoli microgreens, which resulted in delayed postharvest decay of the microgreens. This positive effect of a 10 mM calcium chloride (CaCl_2_) preharvest treatment was confirmed in experiments conducted by Lu et al. [133], which showed a significant increase in aliphatic glucosinolates levels and an overall improvement in visual quality and a longer storage life of broccoli microgreens.

The above examples have shown that applying environmental stresses might be a viable approach to enhance the health-promoting qualities of microscale vegetables. However, the most promising type of environmental stress and its intensity require crop-specific research.

### 5.3. Seed Priming and Biostimulants

Seed priming enhances seed germination, seedling growth, plant establishment, and crop performance. Priming techniques include hydro-priming (soaking seeds in water); osmo-priming (soaking seeds in osmotic solutions, such as polyethylene glycol); halo-priming (soaking seeds in sodium and potassium salts); solid matrix priming (mixing seeds with solid or semi-solid material and a specified amount of water); biopriming (coating seeds with beneficial fungi or bacteria); and treatment with plant growth regulators that are incorporated into the priming medium [134,135,136,137].

Seed priming with potassium nitrate (KNO_3_) improves seedling establishment and plant vigor [138]. Sprouts of three *Medicago* species treated with KNO_3_ showed increased total phenolic and flavonoid contents and enhanced antioxidant and antidiabetic activities [93]. The response of KNO_3_ priming was species-specific, with *Medicago intertexta* showing the highest antioxidant and antidiabetic activities, followed by *M. polymorpha* and *M. indicus*.

The phytohormones jasmonic acid and methyl jasmonate (MeJA) (25–250 μM) and the amino acid DL-methionine (1–10 mM) were used as elicitors to enhance the total glucosinolate content of broccoli and radish sprouts [139]. The most effective treatments consisted of 24 h imbibition of seeds in priming solution, followed by exogenous sprays of elicitors on the cotyledons from days 4 to 7 of sprouting. MeJA priming in combination with exogenous sprays of elicitors led to the most significant increases of total glucosinolate content, from 34% to 100% in broccoli sprouts and from 45% to 118% in radish sprouts.

Commercial biostimulants containing beneficial fungi or bacteria promoting plant growth are often recommended as a sustainable strategy to increase plant performance productivity and produce quality under environmental stresses aggravated by climate change [140]. Plant biostimulants are commonly defined as “*Substance(s) and/or micro-organisms whose function is to stimulate natural processes that enhance nutrient uptake, nutrient use efficiency, tolerance to abiotic stress, and crop quality*” [141]. Bioactive molecules in commercial biostimulants enhance the capability of plants to overcome adverse environmental conditions through their action on primary or secondary plant metabolism [142]. In addition, the presence of phytohormones and other secondary metabolites, vitamins, antioxidants, and inorganic nutrients in the extract of biostimulants may affect plant growth and production directly by enhancing plant tolerance against abiotic stresses [143].

The use of exogenous fungal polysaccharide elicitors obtained from the endophytic fungus *Bionectra pityrodes* (race Fat6) enhanced the sprout growth and flavonoid (rutin, quercetin) production of tartary buckwheat (*Fagopyrum tataricum*) [144]. Seed inoculation of common buckwheat (*Fagopyrum esculentum*) with the endophytic bacterium *Herbaspirillum* sp. (isolate ST-B2), isolated from common buckwheat seedling stems, enhanced the growth of sprouts and microgreens, promoted root elongation, and increased sprout and microgreen yields [145]. Soaking common buckwheat seeds in a solution containing *Ecklonia maxima* algae extract, which is known to enhance plant tolerance to abiotic stressors and plant growth, promoted the accumulation of dry matter in sprouts [48]. Buckwheat sprouts grown from seeds soaked in a solution containing nitrophenols, which occur naturally in plants (Biostimulant Asahi SL), and *Pythium oligandrum* oospores, showed a significantly higher level of crude protein [48]. *P. oligandrum* is a common oomycete found in soils worldwide and has a beneficial effect on pathogen control and induces resistance in the host plant [146]. Therefore, using fungal, bacterial, or other elicitors could be an efficient strategy for improving the nutritional and functional quality of sprouts and microgreens. During recent years, the study and use of plant biostimulants has been steadily growing. They may be applied singly or in combination, and there may be synergistic and additive effects of microbial and non-microbial plant biostimulators. Meanwhile, the design and development of the second generation of plant biostimulants are underway with specific modes of action to render agriculture more sustainable and resilient [141].

### 5.4. Biofortification

The biofortification of vegetables with micronutrients that are essential or beneficial to human health, including iodine, iron, zinc, and selenium, can be achieved in soil-less culture systems [115]. Accurate control of microelement concentrations and constant exposure of roots to the fortified nutrient solution without soil interaction can maximize their uptake, translocation, and accumulation in the edible plant parts.

Selenium (Se) is an essential microelement for living organisms and plays a significant role in antioxidant defense. Many studies have been undertaken on the biofortification of plants to produce Se-enriched foods and elicit the production of secondary metabolites that are beneficial to human health [147]. Biofortification with Se is also used to improve the nutritional quality of sprouts and microgreens via an increase in the overall content of bioactive compounds [148,149]. Bioactive compounds, including phenolics, flavonoids, vitamin C, anthocyanin, and antioxidant activity, significantly increased in wheat microgreens biofortified with moderate levels (0.25–0.50 mg/L) of Se [149]. The biofortification of buckwheat microgreens with a combination of Se and iodine increased microgreen yield by 50–70% compared to single applications of Se or iodine [150]. Moreover, the combination treatment of Se and iodine led to synergistic effects regarding Se accumulation (an increase of 50% over Se application alone). On the other hand, this combination treatment reduced iodine accumulation by 50% over iodine application alone.

Puccinelli et al. [148] noted a higher germination index, higher Se content, and higher antioxidant capacity in microgreens of sweet basil (*Ocimum basilicum*) grown hydroponically and supplemented with 4 or 8 mg Se L^−1^ as sodium selenate. Pannico et al. [151] studied the Se biofortification of four microgreen genotypes (coriander—*Coriandrum sativum*; tatsoi—*Brassica rapa* subsp. *narinosa;* and green and purple basil—*Ocimum basilicum*) to produce Se-enriched foods with a high nutraceutical profile in a simple soil-less cultivation system (SCS). They concluded that the optimal Se dose that guarantees effective Se biofortification and improves the content of bioactive compounds was 16 μM in coriander and tatsoi and 8 μM in green and purple basil. A fresh portion (10 g) of coriander and tatsoi microgreens supplemented with 16 μM Se in SCS satisfied 61% and 90% of Se’s recommended dietary allowance (RDA), respectively. A lower Se supplementation of green and purple basil microgreens with 8 μM in SCS was sufficient to supply 133% and 83% of the RDA of Se, respectively, when consuming a 10 g portion of fresh microgreens [151].

The biofortification of broccoli sprouts with selenium nanoparticles (NSePs) did not affect chlorophyll content, total carotenoid, and total phenols content. Still, it enhanced the antioxidant capacity of the treated sprouts [152]. Puccinelli et al. [94] reported that the wild herb species *Rumex acetosa* (garden sorrel)*, Plantago coronopus* (buck’s-horn plantain)*,* and *Portulaca oleracea* (common purslane) are of interest to produce Se-biofortified microgreens. Especially *P. coronopus* showed a strong correlation between the Se concentration in the growth medium and the Se accumulation detected in the microgreens. Furthermore, Se-biofortified *P. coronopus* microgreens also showed the highest chlorophyll and flavonoid contents [94]. Therefore, consuming microgreens of all three wild herb species would benefit human health.

### 5.5. Effect of Light in Controlled Environments

Light is an essential environmental factor that affects the growth and development of plants. The light environment is critical for seed germination, seedling development, photosynthetic productivity, plant metabolism, and the production of secondary metabolites. For the successful production of plants in controlled environments, it is necessary to deliver the required photons to the plant through artificial means, either by redirecting sunlight indoors, employing artificial lights, or adopting a hybrid lighting system [153]. This also applies to the production of sprouts and microgreens, either in greenhouses or in plant factories (indoor vertical farming). Compared to other artificial light sources, such as fluorescent lamps and high-pressure sodium (HPS) lamps, light-emitting diodes (LEDs) offer several advantages, such as high photoelectric conversion efficiency, low radiant heat output, low energy requirement, and long lifespan [113,122,154]. Moreover, the use of LEDs allows the modification of the light spectra and adjusting light intensities, thus facilitating the regulation of plant growth and quality of the produce. A recent, comprehensive review looked at the effects of ultraviolet radiation alone (see Section 5.2) or in combination with visible spectrum LED lighting on the stimulation of bioactive compounds during the growth and shelf life of sprouts, microgreens, and baby leaves and sheds light on the possible modes of action of these elicitors [122].

#### 5.5.1. Effects of Light Intensity, Exposure Time, and Light Sources

In a study involving three *Brassica* microgreens (purple kohlrabi—*Brassica oleracea* var. *gongylodes;* mizuna—*Brassica rapa* var. *japonica*; and mustard—*Brassica juncea*) grown in hydroponic tray systems on multilayer shelves, percent dry weight increased for kohlrabi, mizuna, and mustard microgreens with an increase in light intensity from 105 to 315 µmol·m^−2^·s^−1^ [154]. This effect was independent of the light quality used.

Irradiance levels can also impact the production of secondary metabolites such as carotenoids and glucosinolates. Studies conducted by Kopsell et al. [155] demonstrated that exposure of mustard (*Brassica juncea*) microgreens to high light intensity (463 μmol photons m^−2^ s^−1^) before harvest increased the content of zeaxanthin (2.3-fold) and antheraxanthin (1.5-fold), but decreased β-carotene and neoxanthin levels [155]. Similarly, increasing light intensities led to a significant decrease in the content of total carotenoids of mizuna and mustard microgreens [156]. For kohlrabi, increasing light intensities enhanced the total concentration of anthocyanins compared with those grown under lower light intensities. According to Kopsell et al. [155], plants adjust to different irradiance levels via the xanthophyll cycle. With exposure to high light intensity, violaxanthin is converted to zeaxanthin via antheraxanthin. This process is reversible under low light intensity.

Samuolienė et al. [157] studied the effect of the irradiance intensity of a combination of blue, red, and far-red LED lighting on the growth, nutritional quality, and antioxidant properties of kohlrabi (*Brassica oleracea* var. *gongylodes*, mustard (*Brassica juncea*), red pak choi (*Brassica rapa* var. *chinensis)*, and tatsoi (*Brassica rapa* var.* rosularis*) microgreens. The light intensities ranged from 110 µmol m^−2^ s^−1^ to 545 µmol m^−2^ s^−1^. The best growth and antioxidant activity results were reached with light intensities of 330 and 440 µmol m^−2^ s^−1^. These irradiance levels led to larger leaf surface area, lower content of nitrates, and higher content of anthocyanins, phenols, and antioxidant capacity [157]. When evaluating the effects of LED photon flux density levels of 545, 440, 330, 220, and 110 μmol m^−2^ s^−1^, Brazaitytė et al. [158] noted a crop-/species-specific reaction. The concentrations of various carotenoids in red pak choi (*Brassica rapa* var. *chinensis*) and tatsoi (*Brassica rapa* subsp. *narinosa*) were higher under the illumination of 330–440 μmol m^−2^ s^−1^. In contrast, mustard (*Brassica juncea*) responded best at 110–220 μmol m^−2^ s^−1^.

In line with the observations made by Samuolienė et al. [157] and Brazaitytė et al. [158] regarding the most effective irradiance levels, Harakotr et al. [86] reported similar findings in experiments with five traditional vegetable microgreens, namely water convolvulus (*Ipomoea aquatica*), red holy basil (*Ocimum sanctum*), lemon basil (*O. africanum*), dill (*Anethum graveolens*), and rat-tailed radish (*Raphanus sativus* var. *caudatus*). These authors concluded that an irradiance level of 330 µmol m^−2^ s^−1^ was optimal for microgreen growth and the accumulation of bioactive compounds of water convolvulus, red holy basil, dill, and lemon basil. In addition, this light intensity led to the highest dry weight, total phenolic and flavonoid contents, and free radical scavenging activity [86]. However, rat-tailed radish microgreens did not respond positively to the irradiance level.

Continuous (24 h per day) lighting, either with LED or fluorescent lighting, enhanced the growth and nutritional quality of microgreens of arugula (*Eruca sativa*), broccoli (*Brassica oleracea* var. *italica*), mizuna (*Brassica rapa* var. *nipposinica*), and radish (*Raphanus sativus* var. *radicula*) in growth chambers [159]. The mild oxidative stress induced by continuous light treatment led to an increase in non-enzymatic antioxidants (anthocyanin, flavonoid, and proline) and enhanced the activities of antioxidant enzymes. As the effects were more pronounced under LED lighting, Shibaeva et al. [159] concluded that one could produce microgreens of arugula, broccoli, mizuna, and radish under LED continuous light with economic and nutritional benefits.

In common buckwheat (*Fagopyrum esculentum*), LED lighting in phytotrons stimulated the production of health-promoting phenolic compounds. Still, it led to more compact plants and a decrease in above-ground biomass compared with solar light supplemented with high-pressure sodium (HPS) lamps [160].

When comparing the effect of different light sources (incandescent, fluorescent, and LED RGB (red, green, blue)) on kale sprouts, LED lights resulted in the highest yield and the highest amounts of chlorophylls, β-carotene, lutein, neoxanthin, and ascorbic acid content [161]. Furthermore, compared to incandescent/fluorescent lighting, broccoli microgreens under high proportions of blue (20%) and red (80%) LED lighting accumulated a higher proportion of chlorophylls, carotenoids, glucoraphanin, and minerals [162].

Traditional high-pressure sodium lamps (400 W; 600 nm) supplemented with blue LED light (450 nm) and blue-violet LED light (420 and 440 nm, respectively) under a 24-h photoperiod and a photon flux density of 300 ± 10 μmol m^−2^ s^−1^ increased the production of phenolic acids in basil (*Ocimum basilicum*) plants. In addition, flavonoids were enhanced in arugula (*Eruca sativa*) plants compared to the control (high-pressure sodium lamps only) [163].

#### 5.5.2. Effects of Light Spectra

Blue (400–500 nm) wavelengths promote the photosynthetic process by inducing stomatal opening and chloroplast relocation and enhancing the accumulation of antioxidant compounds and pigments in vegetables and fruits [164]. Blue light also affects vegetative and leaf growth and is particularly important for young plants such as sprouts, microgreens, or baby leaves [122]. Red light (600–700 nm) is another crucial factor that enhances the growth rates of plants and promotes the synthesis of pigments and phytochemicals in different plant species, thus improving the nutritional quality of the product [164]. Red light interacts with blue light in regulating plant responses. When the appropriate light intensity is applied, the optimal, crop-specific R:B ratio enhances photosynthetic capacity and improves growth and yield. Far-red light (700–800 nm) contributes to plant elongation and triggers flowering and fruit production and biomass accumulation in plants [165]. Green light (550 nm) also contributes to photosynthesis and biomass accumulation and may influence secondary metabolism [164].

Recently, Appolloni et al. [166] undertook a review on the effects of LED lighting on the content of phytochemicals in medicinal and aromatic plants, microgreens, and edible flowers. Among the 40 papers reviewed, most studies used a combination of red and blue light (22%) or monochromatic blue light (23%), with a 16 h day^−1^ photoperiod (78%) and a light intensity greater than 200 μmol m^−2^ s^−1^ (77%). The application of red and blue light together, sometimes also in combination with other spectra (far-red and green), often showed beneficial effects regarding the accumulation of phytochemicals, particularly in the case of microgreens [166]. However, the impact of red and blue light on the synthesis of specialized metabolites may vary with the plant species studied.

Sprouts are usually grown in darkness. Compared to darkness, fluorescent light treatments with white, blue, and red at a moderate photon flux density (110 µmoL m^−2^.s^−1^) increased the contents of vitamin C and pigments such as carotenoids, chlorophylls, and anthocyanins in 5-day-old sprouts of radish, soybean, mungbean, and pumpkin [167]. In addition, increased contents of soluble proteins and sugars were observed in soybean and pumpkin sprouts, respectively, exposed to light treatments. However, polyphenols were increased only with soybean sprouts and only with red light applications. Similarly, fluorescent lighting and combined LED lighting treatments of blue (B) + red (R) and B + R + FR (far-red) improved total antioxidant activity. They increased the content of bioactive compounds in carrot (*Daucus carota*) sprouts compared to darkness [127]. Both combined LED treatments increased the phenolic content (phenolic acids and rutin) by 45% and 65%, respectively, compared to darkness, and 32% compared to fluorescent light. Moreover, the combined B + R LED treatment also enhanced the content of carotenoids, but not when far-red was added.

Comparing three light sources, namely fluorescent light lamps, blue (460 mm) LED lights, and red (625 nm) LED lights, at a light intensity of 35 μmol/m^2^ s^−1^, common buckwheat (*Fagopyrum esculentum*) sprouts exhibited the highest total phenolics and total flavonoids contents. Furthermore, they showed the most increased antioxidant activities when grown under blue light [168]. Experiments conducted with rapeseed (*Brassica napus*) sprouts exposed to white (380 nm), blue (470 nm), red (660 nm), and blue + red LED lighting under a 16-h photoperiod and a photon flux density of 50 µmol/s·m^2^, revealed that red LED light enhances sprout growth. In contrast, blue light effectively increased the accumulation of glucosinolates and phenolics [169]. Similarly, phenolics, flavonoids, and glucoraphanin accumulated in broccoli sprouts under a photon flux density of 50 µmol m^−2^ s ^−1^ of blue LED lighting on their own and/or combined with red LED lighting at an equal ratio (50:50) [170].

Basal LED spectra composed of a combination of blue, red, and far-red light and supplemented with green (510 nm) led to a significant increase in the content of total phenols, α-tocopherol, and vitamin C, and enhanced the antioxidant capacity in sprouted seeds of lentil (*Lens esculenta*) and wheat (*Triticum aestivum*) [171]. In addition, basal LED spectra supplemented with amber (595 nm) significantly enhanced the antioxidant properties of radish (*Raphanus sativus*) sprouts.

Like in sprouts, the contents of anthocyanins and phenolic acids are primarily influenced by the proportion of red and blue light in microgreens. Blue and red light LED treatments have been shown to enhance the growth of basil (*Ocimum basilicum*) microgreens and increase phenolic compounds’ contents [172]. However, the effect of light quality on the synthesis of phenolic substances and free radical scavenging activity was cultivar-specific. The green basil cultivar was strongly stimulated by a higher proportion of red light (2R:1B), while the red cultivar showed the best response when exposed to a higher ratio of blue light (1R:2B).

This cultivar-specific reaction of basil microgreens in response to different LED spectra was also reported by Chutimanukul et al. [173]. These authors used different LED light spectra ratios of red^®^, green (G), and blue (B), and assessed their impact on photosynthesis, biomass, antioxidant capacity, and secondary metabolites of green and red holy basil (*Ocimum tenuiflorum*) plants grown hydroponically under a controlled environment. An R:B ratio of 1:3 (high percentage of the blue spectrum) significantly increased the total phenolics content (TPC) and free radical scavenging activity in leaves of both holy basil cultivars [173]. In contrast to TPC, the effect of LED light spectra on total flavonoid content was cultivar-dependent [173]. This example clearly illustrates that the red and blue LED lighting ratios need to be adjusted to the respective cultivar.

In experiments conducted by Brazaitytė et al. [174] with mustard (*B. juncea*) and kale (*B. napus*) microgreens, a high percentage (75 or 100%) of blue light was applied in the mix with red light (B75R25 and B100R0) at 220 µmol m^−2^ s^−1^ in an 18 h photoperiod for 5 days. This treatment positively affected the accumulation of macro- and micronutrients at the expense of a significant yield reduction [174]. Therefore, the authors recommended a lower proportion (25% or 50%) of blue light as a strategic tool for mustard and kale microgreen biofortification to increase chlorophyll, flavonol, anthocyanin, and carotenoid contents while maintaining high yields. Similar observations have been made by Samuolienė et al. [175] with mustard (*Brassica juncea*), beet (*Beta vulgaris* subsp. *vulgaris*), and parsley (*Petroselinum crispum*) microgreens.

The effect of three LED light sources emitting red/blue ratios of about 2, 5, and 9 units (RB2, RB5, and RB9, respectively) was tested on mustard, radish, green basil, red amaranth, garlic chives, borage, and pea microgreens grown in a vertical farming system [91]. The yield was enhanced in mustard, green basil, and pea microgreens when exposed to a high red portion (RB9), while garlic chives exhibited the highest fresh weight under RB2. In addition, RB9 increased the total phenolic content in all microgreens except for mustard, and increased the antioxidant capacity of pea, green basil, borage, red amaranth, and garlic chives.

Hytönen et al. [176] compared the effects of LED lighting with different spectral compositions on the growth, development, and nutritional quality of lettuce (*Lactuca sativa*). The authors were able to show that warm-white and warm-white supplemented with blue spectra provide equal growth and product quality compared to conventional HPS lighting, both in the absence and presence of daylight. Furthermore, their research demonstrated that a red + blue LED spectrum increased the concentration of several vitamins in lettuce but led to a decrease in biomass accumulation when daylight was excluded [176]. The far-red LED component proved to be more critical than green light or the red/blue ratio for biomass accumulation.

Purslane (*Portulaca oleracea*) microgreens grown for 21 days under saline conditions (80 mM of NaCl) had higher yields and lower contents of antinutrients when treated with combined B + R or combined B + R + FR LED lighting at a photon flux density of 150 µmol m^−2^ s^−1^ [177].

Even a short exposure of broccoli microgreens before harvest to blue LED light (41 μmol·m^−2^·s^−1^) significantly increased levels of shoot tissue β-carotene, violaxanthin, total xanthophyll cycle pigments, glucoraphanin, epiprogoitrin, aliphatic glucosinolates, essential micronutrients (Cu, Fe, B, Mn, Mo, Na, Zn), and essential macronutrients (Ca, P, K, Mg, and S) [178]. Hence, preharvest, short-duration blue light treatment effectively enhances broccoli microgreens’ nutritional value and increases health-promoting phytochemical compounds such as carotenoids and glucosinolates.

Alrifai et al. [179] demonstrated that the incorporation of amber light (590 nm) into a combination of standard blue (455 nm) and red (655 nm) LED lights during the growth of four different *Brassica* microgreens improved the glucosinolate profile by modulating the biosynthesis of the precursors of isothiocyanates. The four species used were: mizunas (*Brassica rapa* var. *japonica*), pak choi (*Brassica rapa* var. *chinensis*), red radish (*Raphanus sativus*), and white mustard (*Brassica juncea*). In earlier experiments with identical light spectra and the same microgreen species and cultivars, these authors made similar observations regarding the biosynthesis of phenolic compounds [180]. While increasing amber and blue and concurrently decreasing the proportion of red light, overall positive correlations were observed for total phenolics, total flavonoid contents, and antioxidant activities. Alrifai et al. [180] concluded that the microgreens could be clustered into three groups based on phytochemical contents and sensitivity to the lighting as follows: (i) radish shows a high blue and amber dose-dependence regarding total phenolics and flavonoids content and antioxidant activity; (ii) mustards show a moderate to high sensitivity to overall lighting but no clear dose-dependence; and (iii) mizunas and pak choi show variable responses to lighting.

Experiments with *Brassica* microgreens grown under four different LED ratios (%) showed that supplemental lighting with 70% red, 10% green, and 20% blue (R_70_:G_10_:B_20_) LEDs enhanced vegetative growth, while dominantly blue LEDs (R_20_:B_80_) increased the mineral and vitamin contents [181]. With the aim to balance microgreen yield with nutritional content, the best treatment proved to be the LED combination with green light (R_70_:G_10_:B_20_), which resulted in the highest growth and nutritional content.

The exposure of soybean (*Glycine max*) microgreens to monochromatic blue LED light and ultraviolet-A (UV-A) radiation led to significant increases in total phenolic and total flavonoid content, as compared with the white LED light control [182]. Among four light spectra (100% red, 100% green, 100% blue, and an R:G:B ratio of 1:1:1) tested, flaxseed (*Linum usitatissimum*) microgreens treated with 100% blue light exhibited the highest content of flavonoids (2.48 mg catechin equivalents [CAE] g^−1^ FW), total phenols (3.76 mg GAE g^−1^ FW), chlorogenic acid (1.10 mg g^−1^ FW), and antioxidant capacity (8.06 µmol Trolox equivalent antioxidant capacity [TEAC] g^−1^ FW) [103].

In summary, significant progress has been made in recent years regarding the effects of light intensity, exposure time, light sources and light spectra on plant growth and the development of microscale vegetables in controlled environments. However, further studies are needed to elucidate the crop-specific interactions between light spectrum and light intensity and their relationship with other environmental factors [164].

## 6. Microgreen Market Trends and Outlook

The popularity of microscale vegetables is apparent, judging from their increasing use in upscale restaurants and the steadily growing availability of a wide range of microgreens in supermarkets. Once purchased, they continue actively growing on their media and are ready for cutting and use as perfectly fresh vegetables in homemade meals. Apart from human nutrition, cosmetics is another niche industry that drives the growth of microgreens [183]. Vitamin- and nutrient-dense microgreens are processed into oils and ingredients for skincare products, shampoos, and conditioners, making them attractive for health-conscious consumers.

Sprouts and microgreens are versatile, as they can quickly, easily, and cost-effectively be grown in urban and peri-urban settings where land is often a limiting factor. They can even be produced inside or around residential areas by the consumers themselves, independent of seasonal growth cycles [21,65,184], or through indoor farming (greenhouses and vertical farming) by specialized producers for sale in supermarkets [22,25,26,185]. Indoor farming is independent of arable land and external climate conditions. It can use different growing systems (hydroponics, aeroponics, aquaponics, or soil-based) and structures (glass or poly greenhouses, indoor vertical farms, or low-tech plastic hoop houses) and can be based in urban and peri-urban or rural areas, dependent on convenience for logistics, marketing, and costs [185,186]. Because of climate change discussions and the demand for more local food production to reduce the carbon footprint associated with transportation distances, there is now a clear trend to establish greenhouses and indoor vertical farms near urban and peri-urban areas to bring operations closer to the consumers [187].

The COVID-19 pandemic has accelerated this trend towards microscale vegetables as consumers are eager to strengthen their immune systems by consuming food rich in antioxidants and other health-promoting substances. Moreover, in the face of a global disruption of supply chains and significant changes in shopping habits due to the ongoing COVID-19 pandemic, do-it-yourself sprouts and microgreens offer an exciting and sustainable alternative. Growing sprouts and microgreens at home is easy to put into practice. It eliminates the need for long-distance transport, reduces fossil fuel consumption for product delivery, and provides consumer access to highly fresh, nutrient-dense produce that can be harvested as needed for meal preparation [105]. Local production and consumption of sprouts and microgreens would also reduce food waste partially caused by the food supply chain from farm via wholesale markets and consumer outlets.

A feasibility study on microgreens was conducted in India comprising ten microgreens: carrot, fenugreek, mustard, onion, radish, red roselle, spinach, sunflower, fennel, and French basil [65]. The market value of the microgreens was found to be five to eleven-fold greater than their production costs. Thus, microgreen production represents a viable enterprise that can support the economic stability of the rural and urban poor.

With the growing interest and demand from consumers, the global microgreens market is projected to register an estimated compound annual growth rate ranging from 7.5% during the period from 2021 to 2026 [183] to 13.1% during the period from 2020 to 2028 [188]. North America accounted for the most significant global market share in microgreens in 2020, and the Asia-Pacific region has the fastest-growing microgreens market [183].

Several varieties of the same crop or different crops can be grown together to enhance the diversity of tastes, colors, and textures [21]. They are then marketed as specialty mixes, such as “sweet,” “mild,” “colorful,” or “spicy,” fetching prices of US $66–110 per kg in the U.S. [34].

Based on a 2017 report of the intelligence platform Agrilyst [185], the five main crops grown under indoor farming in the U.S. were leafy greens, microgreens, herbs, flowers, and tomatoes. On average, leafy greens and microgreens had the highest profit margin at 40% across the various facility and system types, flowers reached a profit margin of 30%, and tomatoes only 10% [185]. In 2020, the percentage of the microgreen segment among total crop cultivation in greenhouses in the U.S. varied from 25% in the Midwest to 59% in the Northeast and 71% in the South [183].

Members of the Brassicaceae family (broccoli, cabbage, cauliflower, arugula, radish, and cress) dominated the global microgreen market in 2019 [189]. The top microgreen crops according to their market share in 2019 were: broccoli > arugula > cabbage > cauliflower > others > peas > basil > radish > cress. Hence, only peas (Fabaceae) and basil (Lamiaceae) were able to figure among the top microgreen crop types. This huge share of microgreen crop types of the Brassicaceae family in the global microgreen market can be attributed to the commonly known health benefits associated with Brassicaceae crops, which help fight diet-related NCDs [188]. Consumers usually buy fresh microgreens in retail stores (hypermarkets, supermarkets, or grocery stores), and this sector held a market share of 46.8% in 2019, followed by farmers markets [187]. The retail sector is forecast to grow annually by 11.4% from 2021 to 2028. The global microgreen market is expected to grow by 11.1% annually from 2021 to 2028 [189]. The global microgreens market is projected to reach $3795.47 million by 2028, starting from $1417.64 million in 2020 [188]. Prominent microgreen market trends include continued indoor, vertical, and greenhouse farming growth, a rise in the use of advanced production technologies, and a growing awareness for premium food products [188].

## 7. Conclusions

Sprouts and microgreens are novel functional food sources with great potential for sustainably diversifying global food systems, promoting human health, and facilitating the access of a steadily growing urban population to fresh microscale vegetables. These novel food sources have vivid colors, exciting textures, and diverse flavors and tastes, and they can be purchased in supermarkets or even home-grown for daily harvesting as needed. Furthermore, due to their short growth cycle, these nutrient-dense food sources can be produced with minimal input, without using pesticides; hence, they have low environmental impacts and a broad acceptance among health-conscious consumers. Furthermore, as sprouts and microgreens are usually consumed raw, there is hardly a loss or degradation of heat-sensitive micronutrients or vitamins through food processing.

## Figures and Tables

**Table 1:** The crop groups commonly used for sprouting and microgreen production.

Crop Group	Family	Species	Common Name	Main Use ^1^
Legumes	Fabaceae	*Arachis hypogaea*	peanut	S
		*Cicer arietinum*	chickpea	S & M
		*Glycine max*	soybean	S
		*Lens culinaris*	lentil	S & M
		*Medicago sativa*	alfalfa	S & M
		*Trifolium repens*	clover	S & M
		*Vigna angularis*	adzuki bean	S (& M)
		*Vigna mungo*	black gram	S
		*Vigna radiata*	mungbean	S (& M)
		*Vigna unguiculata*	cowpea	S
Cereals	Poaceae	*Hordeum vulgare*	barley	S & M
		*Zea mays*	maize	S & M
		*Avena sativa*	oat	S &M
		*Oryza sativa*	rice	S & M
		*Secale cereale*	rye	S & M
		*Triticum aestivum*	wheat	S & M
		*Zea mays*	maize, popcorn	S & M
Pseudocereals	Amaranthaceae	*Amaranthus sp.*	amaranth	S & M
		*Chenopodium quinoa*	quinoa	S & M
	Polygonaceae	*Fagopyrum esculentum*	buckwheat	S & M
Oilseeds	Asteraceae	*Helianthus annuus*	sunflower	S & M
	Betulaceae	*Corylus avellana*	hazelnut	S
	Linaceae	*Linum usitatissimum*	linseed, flax	S & M
	Pedaliaceae	*Sesamum indicum*	sesame	S & M
	Rosaceae	*Prunus amygdalus*	almond	S
Vegetables & herbs	Amaranthaceae	*Beta vulgaris*	beet	S & M
		*Spinacia oleracea*	spinach	S & M
	Amaryllidaceae	*Allium cepa*	onion	S & M
		*Allium fistulosum*	spring onion	S & M
		*Allium porrum*	leek	S & M
		*Allium schoenoprasum*	chives	S & M
	Apiaceae	*Apium graveolens*	celery	S & M
		*Coriandrum sativum*	coriander	S & M
		*Daucus carota* subsp. *sativus*	carrot	S & M
		*Foeniculum vulgare*	fennel	S & M
		*Petroselinum crispum*	parsley	S & M
	Asteraceae	*Lactuca sativa*	lettuce	S & M
	Brassicaceae	*Brassica juncea*	purple mustard	S & M
		*Brassica oleracea,* var. *alboglabra*	Chinese kale	S & M
		*Brassica oleracea* var. *capitata*	(red) cabbage	S & M
		*Brassica oleracea* var. *gongylodes*	purple kohlrabi	S & M
		*Brassica oleracea* var. *italica*	broccoli	S & M
		*Brassica rapa* var. *chinensis*	pak choi	S & M
		*Brassica rapa* var.* niposinica*	mizuna	S & M
		*Brassica rapa* var. *rapa*	turnip	S & M
		*Brassica rapa* var. *rosularis*	flat cabbage; tatsoi	S & M
		*Eruca sativa*	arugula, rocket	S & M
		*Lepidium bonariense*	peppercress	S & M
		*Nasturtium officinale*	watercress	S & M
		*Raphanus raphanistrum* subsp. *sativus*	daikon, small radish	S & M
	Fabaceae	*Trigonella foenum-graecum*	fenugreek	S & M
		*Pisum sativum*	garden pea	S & M
		*Pisum sativum var. saccharatum*	snow peas	S & M
	Lamiaceae	*Melissa officinalis*	lemon balm	S & M
		*Ocimum basilicum*	sweet basil	S & M
		*Perilla frutescens*	purple perilla	S & M

^1^ Sprouts (S), Microgreens (M) or both (S & M).
